# Identification of *Phytophthora cinnamomi* CRN effectors and their roles in manipulating cell death during *Persea americana* infection

**DOI:** 10.1186/s12864-024-10358-3

**Published:** 2024-05-02

**Authors:** Kayla A. Midgley, Noëlani van den Berg, Robert Backer, Velushka Swart

**Affiliations:** Hans Merensky Chair in Avocado Research, Department of Biochemistry; Genetics and Microbiology, Forestry and Agricultural Biotechnology Institute, University of Pretoria, Pretoria, 0002 South Africa

**Keywords:** Plant-pathogen interactions, Crinkler effectors, Molecular characterization, *In silico*, Protein prediction, Alleles, *Phytophthora*, Hemibiotroph

## Abstract

The oomycete *Phytophthora cinnamomi* is a devastating plant pathogen with a notably broad host range. It is the causal agent of Phytophthora root rot (PRR), arguably the most economically important yield-limiting disease in *Persea americana* (avocado). Despite this, our understanding of the mechanisms *P. cinnamomi* employs to infect and successfully colonize avocado remains limited, particularly regarding the pathogen’s ability to maintain its biotrophic and necrotrophic lifestyles during infection. The pathogen utilises a large repertoire of effector proteins which function in facilitating and establishing disease in susceptible host plants. Crinkling and necrosis effectors (CRN/Crinklers) are suspected to manipulate cell death to aid in maintenance of the pathogens biotrophic and necrotrophic lifestyles during different stages of infection. The current study identified 25 *P. cinnamomi CRN* effectors from the GKB4 genome using an HMM profile and assigned putative function to them as either cell death inducers or suppressors. Function was assigned to 10 PcinCRNs by analysing their RNA-seq expression profiles, relatedness to other functionally characterised *Phytophthora* CRNs and tertiary protein predictions. The full-length coding sequences for these *PcinCRNs* were confirmed by Sanger sequencing, six of which were found to have two divergent alleles. The presence of alleles indicates that the proteins encoded may perform contradicting functions in cell death manipulation, or function in different host plant species. Overall, this study provides a foundation for future research on *P. cinnamomi* infection and cell death manipulation mechanisms.

**Supplementary Information:**

The online version contains supplementary material available at 10.1186/s12864-024-10358-3.

## Background

*Phytophthora cinnamomi* (Rands.) is a soil-borne, hemi-biotrophic oomycete. This pathogen is most often associated with root rot diseases, interfering with water uptake and transport to shoots, which subsequently causes leaf wilting, chlorosis and plant death [1]. Due to the pathogens’ extensive host range (known to infect more than 5000 plant species) [1], *P. cinnamomi* is regarded as one of the most devastating plant pathogens worldwide and causes significant losses to both agricultural and forestry crops with the most significant food losses occurring in avocados (*Persea americana* (Mill.)) [2–8]. The pathogen is also known for damaging the environment and impeding attempts to mitigate climate change, where diseases caused by *P. cinnamomi* could become more severe in regions where the pathogen is already present [3–6, 9, 10].

Despite the economic and ecological relevance of this pathogen, the mechanisms *P. cinnamomi* utilizes to infect and successfully colonize host plants are still largely unknown. In particular, there is little to no knowledge on how *P. cinnamomi* maintains a biotrophic lifestyle early in infection, or switches to a necrotrophic lifestyle later during infection [11]. A likely mechanism utilized to promote biphasic infection would be suppression of the hypersensitive response (HR) during the biotrophic phase and subsequent promotion during the necrotrophic phase [11–14]. The HR is a specialized form of programmed cell death (PCD), involving rapid localized cell death at the site of pathogen penetration and is often associated with disease resistance [15, 16]. This phenomenon can however benefit either the pathogen or the host plant, depending on the lifestyle the pathogen evolves [17]. *P. cinnamomi*, like other *Phytophthora* spp., has likely developed strategies to ‘hijack’ the host plant’s cell death machinery/pathway, causing HR suppression or induction at inappropriate stages of infection [3, 13–15, 18, 19]. This could be accomplished through the differential expression and delivery of cell death-manipulating effectors at different infection stages. During the necrotrophic phase of the pathogen, effector proteins that promote cell death would be expressed, while effectors that suppress cell death would be expressed during the biotrophic phase. The functional characterization of *Phytophthora* effectors has revealed numerous effectors that function in the manipulation of HR [3, 14].

One class of *Phytophthora* effectors that have been repeatedly implicated in cell death suppression and induction are the crinkling and necrosis effectors (CRN/Crinklers). In *Phytophthora* spp., *CRNs* are composed of large multi-gene families that encode cytoplasmic effector proteins [20]. CRN protein sequences possess a highly conserved N-terminal domain containing the LXLFLAK and HVLVXXP motifs, followed by a variable C-terminal. These effectors were originally identified by their ability to induce crinkling and necrosis in plant tissue, but research has revealed CRNs also function in targeting host factors to suppress plant defenses and play important roles in cell death [12, 13, 18–24]. A dual RNA-sequencing (RNA-seq) experiment of the compatible interaction between *Eucalyptus nitens* and *P. cinnamomi* found that the most abundantly expressed *P. cinnamomi* gene was a putative *CRN* effector [25]. This same CRN from *P. cinnamomi* was found to be closely related to a cell death inducing CRN from *Phytophthora infestans* (CRN1), suggesting that the *P. cinnamomi* CRN may play a similar role [20].

Some *Phytophthora* spp. have been shown to have at least two CRNs with contradicting functions - where one suppresses and the other induces cell death - with both effectors being essential for virulence [11, 12, 18]. Computational and functional genomic approaches were used to study two *Phytophthora sojae* CRN effectors - PsCRN63 and PsCRN115 [18]. This work was later supplemented with characterization using *Agrobacterium tumefaciens* infiltration assays in *Nicotiana benthamiana* to reveal the function of these PsCRNs. The study found that PsCRN63 induced cell death and PsCRN115 suppressed cell death while subsequent silencing of one or both *PsCRN*s revealed that both were required for virulence. Similar results were found in PpCRN7 and PpCRN20 from *Phytophthora parasitica* [12]. *A. tumefaciens* infiltration assays in *N. benthamiana* were also conducted using these two PpCRNs, which showed that PpCRN7 increased HR through an additive effect while PpCRN20 suppressed HR. Despite the contradicting functions of PpCRN7 and PpCRN20 in cell death, both effectors were found to increase *N. benthamiana* susceptibility to *P. parasitica*. These examples indicate there is a complex relationship between *Phytophthora* CRNs and the cell death pathways within host plant cells, making them important targets for further research.

Our research identified *P. cinnamomi* CRN (PcinCRN) effectors and assigned putative functions in cell death manipulation during *P. americana* infection. PcinCRNs were identified by searching the *P. cinnamomi* GKB4 genome using a Hidden Markov model (HMM). Putative functions were assigned through analyzing *PcinCRN* expression profiles during *P. americana* infection, Sanger sequencing data, phylogenetic comparison to other functionally characterized *Phytophthora* CRNs and protein folding predictions. This study identified 10 PcinCRNs with putative roles in cell death manipulation, and *PcinCRN* divergent alleles that provide contradicting evidence to functions in cell death manipulation.

## Results

### Identification and validation of full-length PcinCRN effectors

**Fig. 1:**
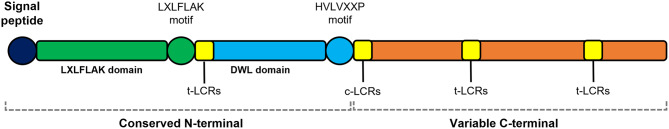
Schematic of a ‘true’ *Phytophthora* CRN effector protein. The characteristic *Phytophthora cinnamomi* crinkling and necrosis (PcinCRN) architecture includes a highly conserved N-terminal with two conserved motifs (LXLFLAK and HVLVXXP), within the LXLFLAK and DWL domains which function in the translocation of the effector from the apoplast into the host plants’ cytoplasm. This is followed by a variable C-terminal that conveys various functions. CRNs do not always contain a signal peptide due to the existence of alternative secretion pathways. Yellow regions indicate regions where terminal and central low complexity regions (t-LCRs and c-LCRs, respectively) can be found. Figure adapted from Midgley et al. (2022) [14]

A repertoire of 25 PcinCRN effectors were identified and validated as ‘true’ PcinCRN effector proteins (Table 1; Fig. 1) - by the presence of two conserved motifs in the N-terminal (LXLFLAK and HVLVXXP) and the absence of a transmembrane helix (TMH) – out of a list of 46 putative PcinCRNs generated from the *P. cinnamomi* GKB4 transcriptome (Supplementary Tables 1 and 2). A partial/CRN-like sequence (PcinCRNpartial1) was also identified from the list of putative PcinCRNs - but was excluded from subsequent analyses. A phylogenetic analysis revealed that all 25 PcinCRNs had similarity to CRNs from other *Phytophthora* spp. with posterior probabilities > 0.5, supporting their designation as ‘true’ CRNs (Supplementary Fig. 1).

**Table 1:** List of 25 full-length PcinCRN effector proteins. The crinkling and necrosis (PcinCRN) effector architecture of all 25 PcinCRNs identified and validated as ‘true’ full-length *Phytophthora cinnamomi* CRN effector proteins. A PcinCRN was validated as a ‘true’ *Phytophthora* CRN if the sequence contained both the LXLFLAK and HVLVXXP motifs and did not contain a trans membrane helix (TMH). The presence/absence of a signal peptide, TMH, low complexity regions (LCR’s), LXLFLAK and HVLVXXP motif are indicated

Sequence ID	Signal peptide	TMH	LXLFLAK motif	HVLVXXP motif	Low Complexity regions (LCR’s)
PcinCRN11	Yes	No	Yes^1^	Yes	Central & terminal LCR
PcinCRN25	Yes	No	Yes^1^	Yes	Terminal LCR
PcinCRN29	Yes	No	Yes^1^	Yes^2^	None
PcinCRN30	Yes	No	Yes	Yes	Central LCR
PcinCRN31	Yes	No	Yes	Yes^2^	Terminal LCR
PcinCRN33	No	No	Yes	Yes^1^	Central LCR
PcinCRN35	Yes	No	Yes^1^	Yes^1^	None
PcinCRN47	No	No	Yes	Yes^1^	Central & terminal LCR
PcinCRN50	Yes	No	Yes^1^	Yes	Terminal LCR
PcinCRN51	No	No	Yes^1^	Yes	None
PcinCRN52	No	No	Yes	Yes	Central LCR
PcinCRN53	No	No	Yes	Yes	None
PcinCRN56	No	No	Yes	Yes^2^	Central & terminal LCR
PcinCRN57	No	No	Yes^1^	Yes^1^	None
PcinCRN73	Yes	No	Yes	Yes^1^	None
PcinCRN74	No	No	Yes	Yes	Central LCR
PcinCRN75	Yes	No	Yes	Yes	None
PcinCRN77	Yes	No	Yes	Yes^1^	None
PcinCRN79	Yes	No	Yes	Yes	None
PcinCRN81	Yes	No	Yes	Yes^2^	Central & terminal LCR
PcinCRN83	Yes	No	Yes	Yes^2^	None
PcinCRN86	Yes	No	Yes^1^	Yes^1^	Terminal LCR
PcinCRN87	Yes	No	Yes	Yes	Terminal LCR
PcinCRN90	Yes	No	Yes	Yes^2^	None
PcinCRN95	No	No	Yes	Yes	Central LCR

^1^The motif differs by a single amino acid; ^2^ The sequence was manually annotated in Integrated Genome Viewer 2.7.2

**Fig. 2:**
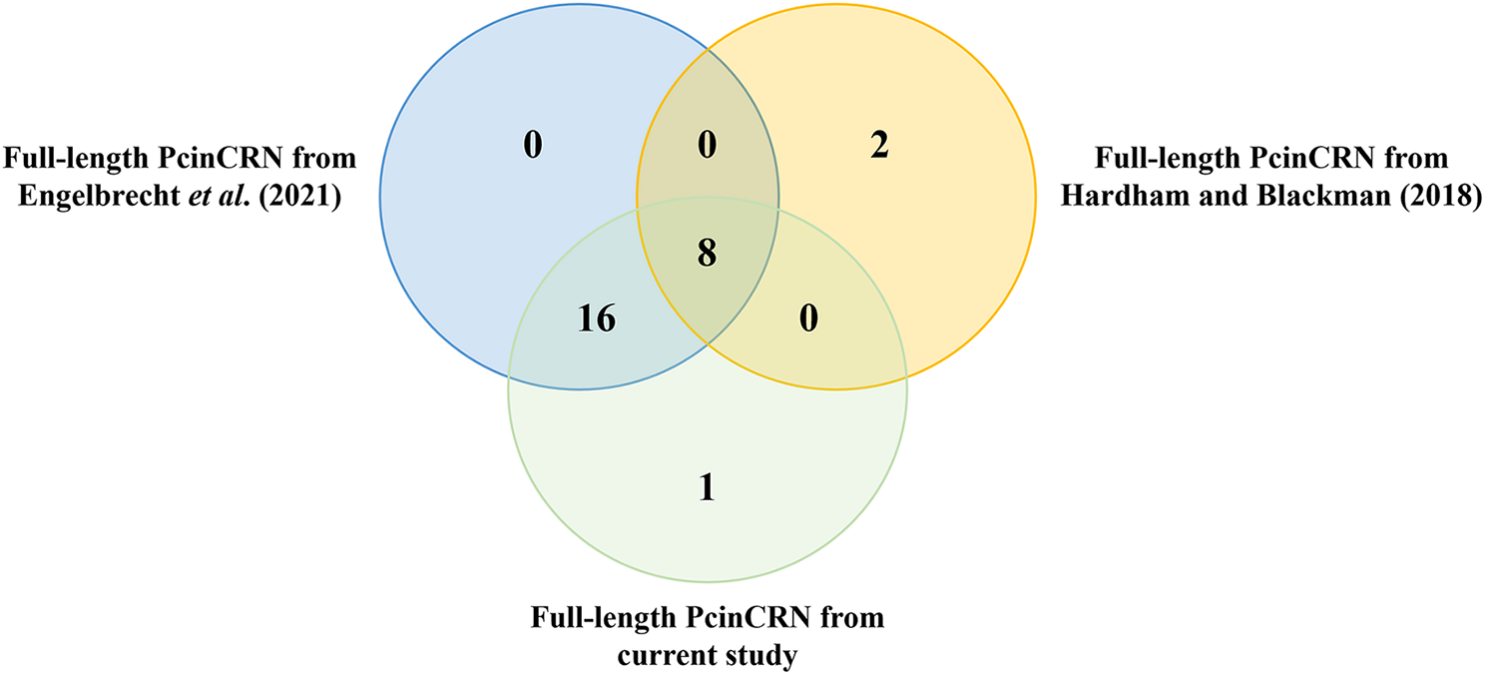
Comparison of *Phytophthora cinnamomi* CRN repertoires from three studies. Venn diagram illustrating the comparison of full-length ‘true’ *Phytophthora cinnamomi* crinkling and necrosis (PcinCRN) effectors identified from Hardham and Blackman (2018) [1], Engelbrecht et al. (2021) [26], and the current study. There are 10 ‘true’ PcinCRNs identified from Hardham and Blackman (2018) [1], 24 identified from Engelbrecht et al. (2021) [26] and 25 identified from the current study. Eight PcinCRNs from Hardham and Blackman (2018) [1] are shared among the PcinCRNs identified by Engelbrecht et al. (2021) [26] and the current study. Sixteen PcinCRNs from Engelbrecht et al. (2021) [26] were among the PcinCRNs identified from the current study. The current study identified one unique PcinCRN

The repertoire generated in this study was compared to the results of putative PcinCRN identified by Hardham and Blackman [1] and Engelbrecht *et al* [26]. in previous studies (Fig. 2, Supplementary Table 3A and 3B). Hardham and Blackman [1] identified 49 putative PcinCRN sequences; but we validated only 10 as ‘true’ PcinCRN effectors and two as partial/CRN-like sequences. Eight of these ‘true’ PcinCRNs were present in the current studies PcinCRN repertoire. Additionally, we determined that twenty-four of the 49 putative PcinCRN sequences identified by Engelbrecht *et al* [26]. were ‘true’ PcinCRN effectors. All ‘true’ PcinCRNs from Engelbrecht *et al* [26]. were present in the repertoire identified by the current study.

### Expression analyses of *PcinCRNs* during infection of avocado

**Fig. 3:**
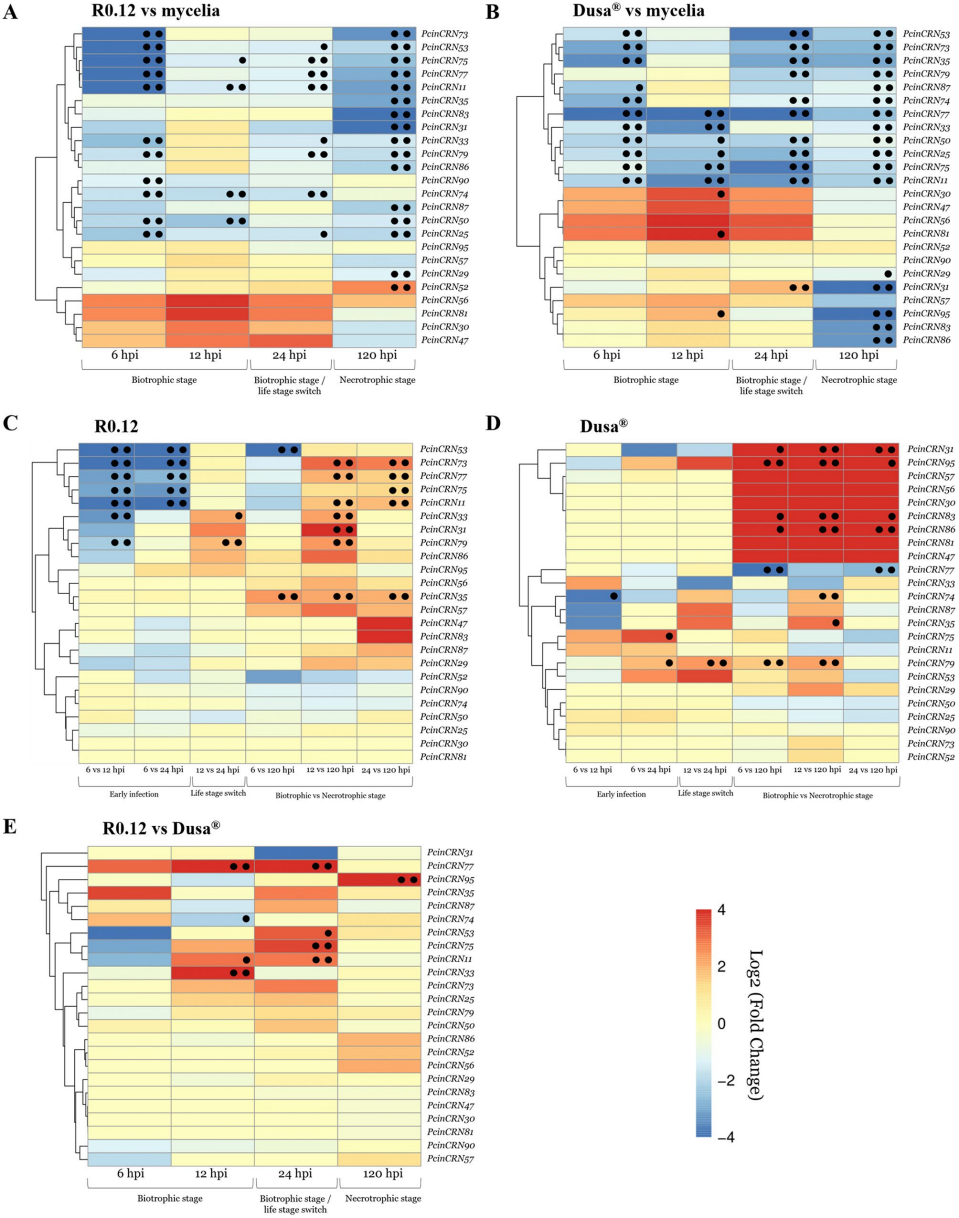
Heatmap depicting the expression of *PcinCRNs* during infection of R0.12 and Dusa® by *Phytophthora cinnamomi.* (**A**) *PcinCRN* expression during infection of R0.12 compared to mycelia. (***B***) *PcinCRN* expression during infection of Dusa® compared to mycelia. (**C**) Comparison of *PcinCRN* expression at different time points during infection of R0.12. (**D**) Comparison of *PcinCRN* expression at different time points during infection of Dusa®. (**E**) Comparison of *PcinCRN* during infection of R0.12 was compared (in-compatible interaction) to the expression during infection of Dusa® (compatible interaction). Expression was compared at 6-, 12-, 24- and 120 hpi. The late biotrophic stage or the possible time-point where the pathogen switches over to the necrotrophic stage is considered as 24 hpi. The necrotrophic stage occurs at 120 hpi. Differential expression was visualised using Log_2_ (Fold Change) and significant differentially expressed genes (DEGs) were identified as those with a Log_2_ (Fold Change) ≥ 1 or ≤-1. Statistical significance was determined using the Benjamini-Hochberg false discovery rate (FDR) method and applying significance cut-off’s (adjusted *p*-value) of < 0.1 (denoted by a single black dot) and < 0.05 (denoted by two black dots)

An RNA-seq analysis was performed to determine what subset of *PcinCRNs* are differentially expressed at the biotrophic or necrotrophic stages during infection of different avocado rootstocks. During infection of the susceptible avocado rootstock (R0.12), the expression of *PcinCRNs* genes were compared to mycelia at 6-, 12-, 24-, and 120 hpi (Fig. 3A, Supplementary Table 4). In comparison to mycelia, 18 *PcinCRNs* were significantly differentially expressed at one or more time points. Nine of these *PcinCRNs* were significantly downregulated during the biotrophic (6-, 12-, and 24 hpi) and necrotrophic (120 hpi) stages compared to mycelia. Differentially expressed genes were denoted using a single black dot (adjusted *p*-value of < 0.1) or two black dots (adjusted *p*-value of < 0.05). *PcinCRN29, PcinCRN31, PcinCRN35, PcinCRN83, PcinCRN86* and *PcinCRN87* were significantly downregulated by more than 4-fold (*p*adj < 0.05) only during the necrotrophic stage compared to the mycelial control. *PcinCRN74* expression was significantly downregulated by more than 3-fold (*p*adj < 0.05) only during the biotrophic phase at 6-, 12-, and 24 hpi. *PcinCRN90* expression was significantly 3-fold downregulated (*p*adj < 0.05) compared to mycelial expression, during the early stage of the biotrophic stage at 6 hpi During the necrotrophic stage, *PcinCRN52* expression was 5-fold upregulated (*p*adj < 0.05) compared to the mycelial control. The RNA-seq expression data for four *PcinCRNs* (*PcinCRN74, PcinCRN79, PcinCRN90*, and *PcinCRN95*) were validated using RT-qPCR, at 12 and 24 hpi (Supplementary Fig. 2).

During infection of the partially resistant avocado rootstock (Dusa®), the expression of *PcinCRNs* genes were compared to mycelia at 6-, 12-, 24-, and 120 hpi (Fig. 3B, Supplementary Table 5). A total of 19 *PcinCRNs* were found to be significantly differentially expressed at one or more time points compared to mycelia. Twelve of these *PcinCRNs* were significantly downregulated during the biotrophic (6-, 12-, and 24 hpi) and necrotrophic (120 hpi) stages compared to mycelia. *PcinCRN29, PcinCRN83*, and *PcinCRN86* were significantly downregulated by more than 2-fold (*p*adj < 0.10 and < 0.05, respectively) only during the necrotrophic stage compared to the mycelial control. The expression of *PcinCRN30* and *PcinCRN81* was upregulated by 20 and 40-fold, respectively (*p*adj < 0.10), during the biotrophic stage at 12 hpi compared to the mycelial control. *PcinCRN31* and *PcinCRN95* were both upregulated by more than 5-fold (*p*adj < 0.10 and < 0.05, respectively) during the biotrophic stage compared to mycelia respectively and were subsequently downregulated by more than 35-fold during the necrotrophic stage compared to mycelia.

*PcinCRN* expression during infection of susceptible R0.12 was compared over time (6-, 12-, 24- and 120 hpi) (Fig. 3C, Supplementary Table 6). In comparison to other time points, nine *PcinCRNs* were significantly differentially expressed at one or more time points. During the biotrophic stage, the expression of eight *PcinCRNs* (*PcinCRN11, 31, 33, 35, 73, 75, 77*, and *79*) increased by more than 2-fold (*p*adj < 0.05) compared to the necrotrophic stage (6-, 12- and 24 hpi compared to 120 hpi). Six PcinCRNs (*PcinCRN11, 33, 73, 75, 77*, and *79*) upregulated during 12- and 24 hpi (compared to 120 hpi) of the biotrophic stage were found to be downregulated by more than 4-fold (padj < 0.05) during the earliest stage of infection at 6 hpi (compared to 12- and 24 hpi).

*PcinCRN* expression during infection of partially resistant Dusa® was also compared over time (Fig. 3D). Seven *PcinCRNs* (*PcinCRN31, 35, 74, 79, 83, 86* and *95*) were significantly upregulated more than 3-fold during the biotrophic stage compared to the necrotrophic stage (*p*adj < 0.1 and < 0.05). One *PcinCRNs* expression (*PcinCRN74*) that was upregulated at 12 hpi (compared to 120 hpi) during the biotrophic stage was downregulated by 14-fold (padj < 0.1) during early infection at 6 hpi (compared to 12- and 24 hpi). *PcinCRN75* and *PcinCRN79* expression was increased by more than 3-fold during the early infection stage (6- vs. 12 hpi, *p*adj < 0.1). During the biotrophic stage, the expression of *PcinCRN77* was significantly reduced by more than 6-fold compared to the necrotrophic stage (*p*adj < 0.05).

Expression of *PcinCRNs* during infection of R0.12 (incompatible interaction) was compared to their expression during infection of Dusa® (compatible interaction) (Fig. 3E, Supplementary Table 7). Expression of *PcinCRN11, PcinCRN33, PcinCRN53, PcinCRN75* and *77* were increased by more than 7-fold in R0.12 compared to Dusa® during the biotrophic stage (*p*adj < 0.1 and < o.o5). *PcinCRN74* expression was decreased by 4-fold during the biotrophic stage in R0.12 compared to Dusa® (*p*adj < 0.1).

Consistent with the known roles of CRN effectors as inducers or suppressors of cell death [12, 13, 18–24]; this RNA-seq analysis identified 13 *PcinCRNs* as putative cell death manipulators. Of the 13, 12 demonstrated the expression patterns of a cell death suppressor (*PcinCRN11, PcinCRN30, PcinCRN31, PcinCRN33, PcinCRN53, PcinCRN73, PcinCRN75, PcinCRN77, PcinCRN81, PcinCRN83, PcinCRN86* and *PcinCRN95*) and only one as a cell death inducer (*PcinCRN52*). (Supplementary Tables 4–8).

### Confirmation of the full-length coding sequences of putative cell death manipulating *PcinCRNs*

Sanger Sequencing was used to sequence *PcinCRN* cDNA in order to confirm their coding sequences for subsequent analyses. The full-length coding sequence of 10 *PcinCRNs* were confirmed following Sanger sequencing of *P. cinnamomi* cDNA. (Table 2, Supplementary Table 9). The sequencing data demonstrated that the first 150 base pairs following the start codon of *PcinCRN77* differed from the original genome assembly annotation. Further analysis using GenomeView 2250 (GV) revealed that the error was likely due to the incorrect assembly of sequencing reads in this region. *PcinCRN77* was the only candidate *PcinCRN* whose nucleotide sequence differed from the original genome annotation, resulting in amino acid sequence variation. Although the amino acid sequence of PcinCRN77 was altered, both conserved CRN motifs (LXLFLAK and HVLVXXP) were present.

**Table 2:** Gene structure of confirmed full-length *PcinCRNs*. The full-length coding sequence was obtained for 10 *Phytophthora cinnamomi* crinkling and necrosis (*PcinCRN*) effector genes via Sanger sequencing of *P. cinnamomi* cDNA pooled from RNA isolated during infection of a susceptible *Persea americana* rootstock (R0.12) at 6, 12, 24 hpi. These data were compared to original genome annotation of the candidate *PcinCRNs* [26]. Six *PcinCRNs* had divergent alleles, of which the single nucleotide polymorphisms (SNPs), consecutive nucleotide substitutions and deletions between the alleles are indicated. *PcinCRN11* underwent alternative splicing. The presence of a nuclear localisation signal (NLS) for each PcinCRN was determined via NLStradamus using a 4 state HMM static model with a Posterior cut-off of 0.3

PcinCRN ID	Number of introns	Consecutive nucleotide substitutions	SNPs	INDELs	Divergent Alleles	NLS
*PcinCRN11*	1	N/A	N/A	NO	1*	NO
*PcinCRN30*	0	N/A	1	NO	2	NO
*PcinCRN52*	1	N/A	N/A	NO	1	YES
*PcinCRN53*	1	N/A	9	YES	2	YES
*PcinCRN73*	0	7	5	YES	2	NO
*PcinCRN75*	0	19	15	YES	2	NO
*PcinCRN77*	0	N/A	N/A	NO	1	NO
*PcinCRN81*	0	N/A	1	NO	2	NO
*PcinCRN86*	0	N/A	N/A	NO	1	NO
*PcinCRN95*	0	N/A	11	NO	2	NO

SNPs: Single nucleotide polymorphisms; INDELS: Insertion or deletion; NLS: Nuclear localization signal; * Alternative splicing occurs in one of the alleles, resulting in the translation of a different protein sequence

**Fig. 4:**
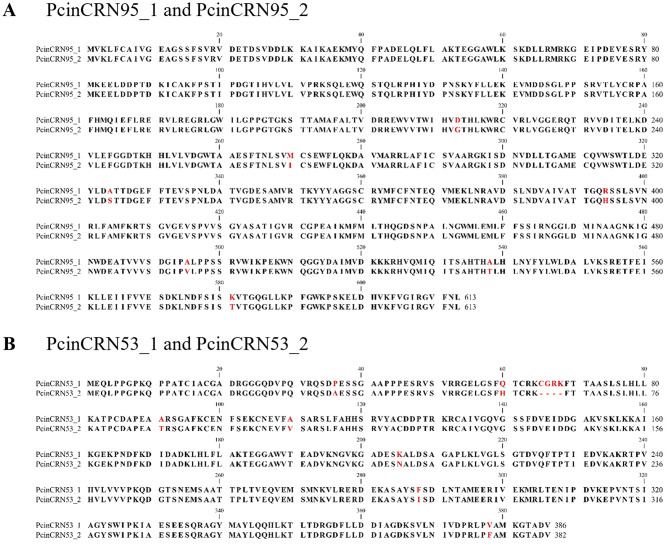
Protein sequence alignment of amino acid sequences translated from alleles of PcinCRN95 and PcinCRN53. The confirmed amino acid sequences of the *Phytophthora cinnamomi* crinkling and necrosis (PcinCRN) effectors of (**A**) PcinCRN95_1 and PcinCRN95_2, and (**B**) PcinCRN53_1 and PcinCRN53_2 were aligned using CLC Main Workbench using default parameters. *PcinCRN95_1* and *PcinCRN95_2* have 11 single nucleotide polymorphisms (SNPs) between them, with seven SNPs resulting in non-synonymous amino acid changes. *PcinCRN53_1* and *PcinCRN53_2* have nine single nucleotide polymorphisms (SNPs) between them, with seven SNPs resulting in non-synonymous amino acid changes. There is a deletion of 12 nucleotides in *PcinCRN53_2* which results in the deletion of a cysteine, glycine, arginine, and lysine from this region compared to PcinCRN53_1. The amino acids highlighted in red indicate the non-synonymous amino acid changes (Supplementary Table 9). Both alleles were confirmed in two additional *P. cinnamomi* isolates (Pcin_isolate129 and Pcin_isolate308) (Supplementary Table 10)

**Fig. 5:**
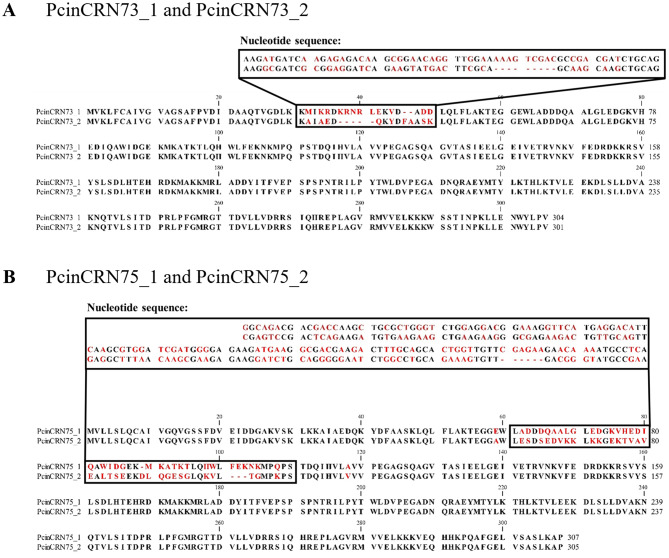
Protein sequence alignment of amino acid sequences translated from alleles of PcinCRN73 and PcinCRN75. The confirmed amino acid sequences of the *Phytophthora cinnamomi* crinkling and necrosis (PcinCRN) effectors of (**A**) PcinCRN73_1 and PcinCRN73_2, and (**B**) PcinCRN75_1 and PcinCRN75_2 were aligned using CLC Main Workbench using default parameters. The black box in the figure shows the nucleotide changes between alleles which results in the region of non-synonymous amino acid changes and deletions indicated in the final protein sequence. The alleles *PcinCRN73_1* and *PcinCRN73_2* have five single nucleotide polymorphisms (SNPs) between them. Numerous consecutive nucleotide substitutions of two or more nucleotides occur throughout the region, resulting in non-synonymous amino acid changes. There is a deletion of nine nucleotides in *PcinCRN73_2* which results in a shifted open reading frame (ORF) as well as amino acid deletions in this region. *PcinCRN75_1* and *PcinCRN75_2* have 15 single nucleotide polymorphisms (SNPs) between them. Numerous consecutive nucleotide substitutions of 2 or more nucleotides occur throughout the region, resulting in non-synonymous amino acid changes. There is a deletion of six nucleotides in *PcinCRN75_2* which results in a deletion of two amino acids in this region. The amino acids highlighted in red indicate the non-synonymous amino acid changes as well as the deletion of amino acids in PcinCRN73_2 (Supplementary Table 9). Both alleles were confirmed in two additional *P. cinnamomi* isolates (Pcin_isolate129 and Pcin_isolate308) (Supplementary Table 10)

Sequencing results revealed two divergent alleles in the *P. cinnamomi* GKB4 genome sequence for six of the *PcinCRN* candidates (*PcinCRN30, PcinCRN53, PcinCRN73, PcinCRN75, PcinCRN81* and *PcinCRN95*). Alleles for *PcinCRN30* and *PcinCRN81* were constituted by a single nucleotide polymorphism (SNP), which resulted in a single non-synonymous amino acid change (Supplementary Fig. 3). *PcinCRN95* had a total of 11 SNPs, seven of which resulted in non-synonymous amino acid changes (*PcinCRN95_1* and *PcinCRN95_2*) (Fig. 4A). The sequence of *PcinCRN53* contained nine SNPs, seven of which resulted in non-synonymous amino acid changes (*PcinCRN53_1* and *PcinCRN53_2*) (Fig. 4B). Additionally, *PcinCRN53* had a 12 bp deletion that resulted in the deletion of a cysteine, glycine, arginine, and lysine from this region. Non-synonymous amino acid changes between alleles were not only the result of SNPs and nucleotide deletions, alleles of *PcinCRN73* and *PcinCRN75* demonstrated consecutive nucleotide substitutions (Fig. 5).

**Fig. 6:**
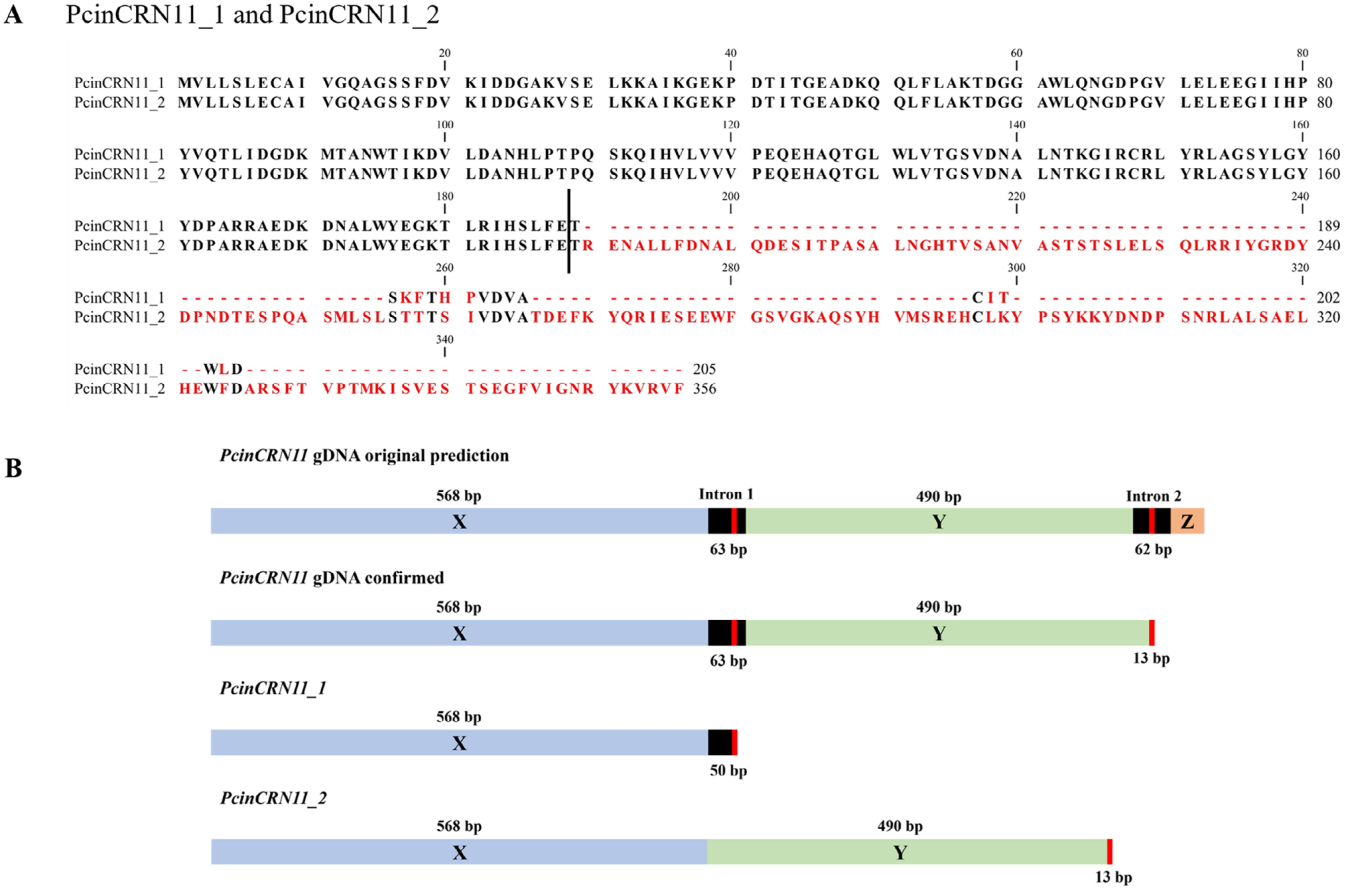
Protein sequence alignment and gene structure of PcinCRN11_1 and PcinCRN11_2. (**A**) The confirmed amino acid sequences of the *Phytophthora cinnamomi* crinkling and necrosis (PcinCRN) effectors PcinCRN11_1 and PcinCRN11_2 were aligned using CLC Main Workbench using default parameters. The black vertical line represents where the site of alternative splicing occurs, and the red amino acids represent amino acid changes because of this altered splicing. (**B**) Diagram illustrating the original full-length *PcinCRN11* gene prediction and the newly confirmed full-length gene sequence. The blue block labelled X and green box labelled Y represents the different exons within the transcribed region. The orange box labelled Z represents an additional exon which was thought to be in the original *PcinCRN11* gene prediction. The black boxes represent introns, and the red line within the introns represent stop codons. Splicing does not occur in *PcinCRN11_1*, resulting in the inclusion of the intron containing a stop codon. The intron is spliced out of PcinCRN11_2 allowing for the inclusion of both exon X and Y

Evidence of intron retention was discovered, which produced a variant of *PcinCRN11_1*; *PcinCRN11_2* (Fig. 6). The cDNA sequence of *PcinCRN11_1* showed no evidence of intron splicing, with the coding sequence consisting of the first of two exons and an intron containing a termination site. However, *PcinCRN11_1* is alternatively spliced to remove the intron, resulting in *PcinCRN11_2* to include both exons (Fig. 6B).

To confirm the presence of all *PcinCRN* alleles, the gDNA of two additional *P. cinnamomi* isolates (Pcin_isolate129 and Pcin_isolate308) were sequenced. All the alleles for *PcinCRN53, PcinCRN75*, and *PcinCRN95* were indeed present in the genomes of both isolates, while *PcinCRN73_1* was confirmed only in Pcin_isolate129 and *PcinCRN73_2* was confirmed only in Pcin_isolate308 (Supplementary Table 10).

### Phylogenetic analysis

**Fig. 7:**
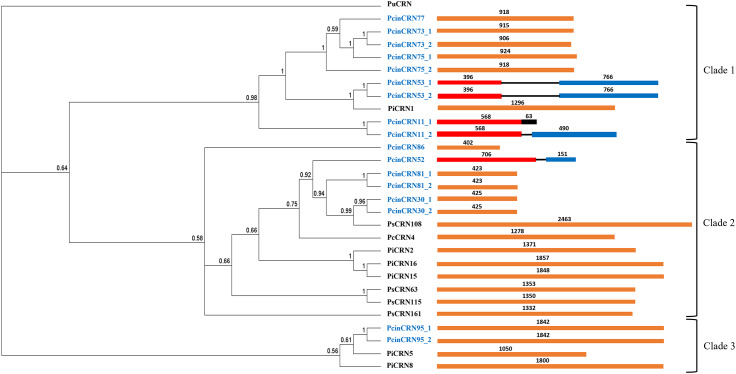
Evolutionary relatedness of full-length PcinCRNs to functionally characterised CRNs from other *Phytophthora* spp. A Phylogenetic tree resulting from Bayesian inference analysis of the confirmed full-length *Phytophthora cinnamomi* crinkling and necrosis (PcinCRNs) effector amino acid sequences aligned with CRNs from other *Phytophthora* spp. (*Phytophthora infestans* CRN, PiCRN; *Phytophthora capsici*, PcCRN and *Phytophthora sojae*, PsCRN) functioning in cell death induction (PiCRN1, PiCRN2, PiCRN5, PiCRN8, PiCRN15, PiCRN16, PcCRN4 and PsCRN63) or suppression (PcCRN108, PsCRN115 and PsCRN161). Support for branches is indicated by posterior probability values, displayed for each node to the second significant digit, with a posterior probability cut-off of < 0.5. A CRN-like protein from *Pythium ultimum* was used as an outgroup (PuCRN, K3WBE4). Three distinct clades were formed. PcinCRNs are denoted in blue while CRNs from other *Phytophthora* spp. are denoted in black. The gene structures for each *CRN* are indicated next to each label. Exons are indicated in red and blue; introns are represented as black lines and genes with no introns are indicated in orange. The black in the gene structure for PcinCRN11_1, represents the predicted intron with an internal stop codon, shown to be retained by sequencing of cDNA. This intron is spliced out in PcinCRN11_2, resulting in two respective proteins of differing length. The numbers above the gene structures indicate the size of the regions in bp

The amino acid sequences of the PcinCRNs were compared to those of CRNs from other *Phytophthora* spp., which had previously been functionally characterised, to gain further evidence towards their putative function. The analysis revealed that the PcinCRNs and other *Phytophthora* CRNs formed three distinct clades (Fig. 7). Each clade is represented by one or more CRNs from other *Phytophthora* spp. with previous functional characterisations as cell death inducers (PiCRN1, PiCRN2, PiCRN5, PiCRN8, PiCRN15, PiCRN16, PcCRN4 and PsCRN63) and/or suppressors (PcCRN108, PsCRN115 and PsCRN161). Clade 1 was comprised of 9 PcinCRNs, grouping within the same clade as a *P. infestans* CRN and this relationship is supported by a posterior probability of 0.98, indicating good support. Clade 2 was comprised of 6 PcinCRNs, grouping within the same clade as three *P. infestans* CRNs, four *P. sojae* CRNs and a *Phytophthora capsici* CRN. This relationship is supported by a posterior probability of 0.58, indicating moderate support. PcinCRN52, was found to be closely related to PcCRN4, but is most similar to PsCRN108. PcinCRN81_1 and PcinCRN81_2 were found to be closely related to PsCRN108. PcinCRN30_1 and PcinCRN30_2 were closely related to PsCRN108. Clade 3 was comprised of two PcinCRNs, grouping within the same clade as two *P. infestans* CRNs and this relationship is moderately supported by a posterior probability of 0.56.

### PcinCRN protein structure prediction

**Fig. 8:**
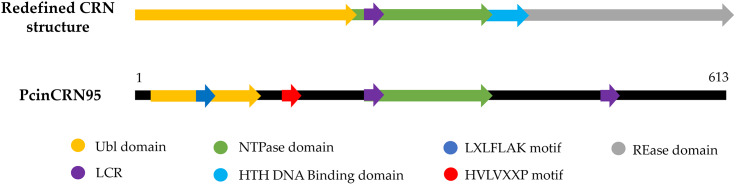
The protein domain architecture of PcinCRN95 closely resembles that of CRNs functioning in cell death [28]. The domains in *Phytophthora cinnamomi* crinkling and necrosis effector protein 95 (PcinCRN95) are compared against the domains present in the CRN architecture of a CRN functioning in the induction of cell death, proposed by Zhang et al. [28]. . It was found that majority of the cell death inducing CRNs possessed a Ubl domain in the N-terminal followed by a NTPase and a restriction endonuclease (REase) superfamily in the C-terminal. The authors suggested that the Ubl domain could facilitate translocation inside the host nucleus, the toxicity function is specified by the REase domain, and the NTPase domain functions in regulating REase activity or affinity toward nucleic acids. There are notable similarities between the PcinCRN95 and the redefined CRN architecture, where both contain a ubiquitin-like (Ubl) domain (yellow), low complexity regions (LCR’s) (purple) and a nucleoside-triphosphatase (NTPase) domain (green) providing evidence for a potentially conserved functional role

Domain analyses were conducted to gain additional evidence toward the assignment of putative functions to PcinCRNs during avocado infection. Analyses of confirmed full-length PcinCRN amino acid sequences revealed that six PcinCRNs (PcinCRN11, PcinCRN 30, PcinCRN52, PcinCRN81, PcinCRN86 and PcinCRN95) possessed one or more low complexity regions (LCR’s) (Table 1), and all PcinCRNs except PcinCRN30, PcinCRN53_2 and PcinCRN86 contained one or more CRN domains as described by Haas et al. (2009) [27] (Supplementary table X11. PcinCRN30, PcinCRN52 and PcinCRN95 contained a central LCRs, PcinCRN86 had a terminal LCR and both PcinCRN11 and PcinCRN81 contained both a central and terminal LCR. A ubiquitin-like (Ubl) domain and a phosphate-loop (P-loop) nucleoside-triphosphatase domain (NTPase) were identified within PcinCRN95 (Fig. 8).

**Fig. 9:**
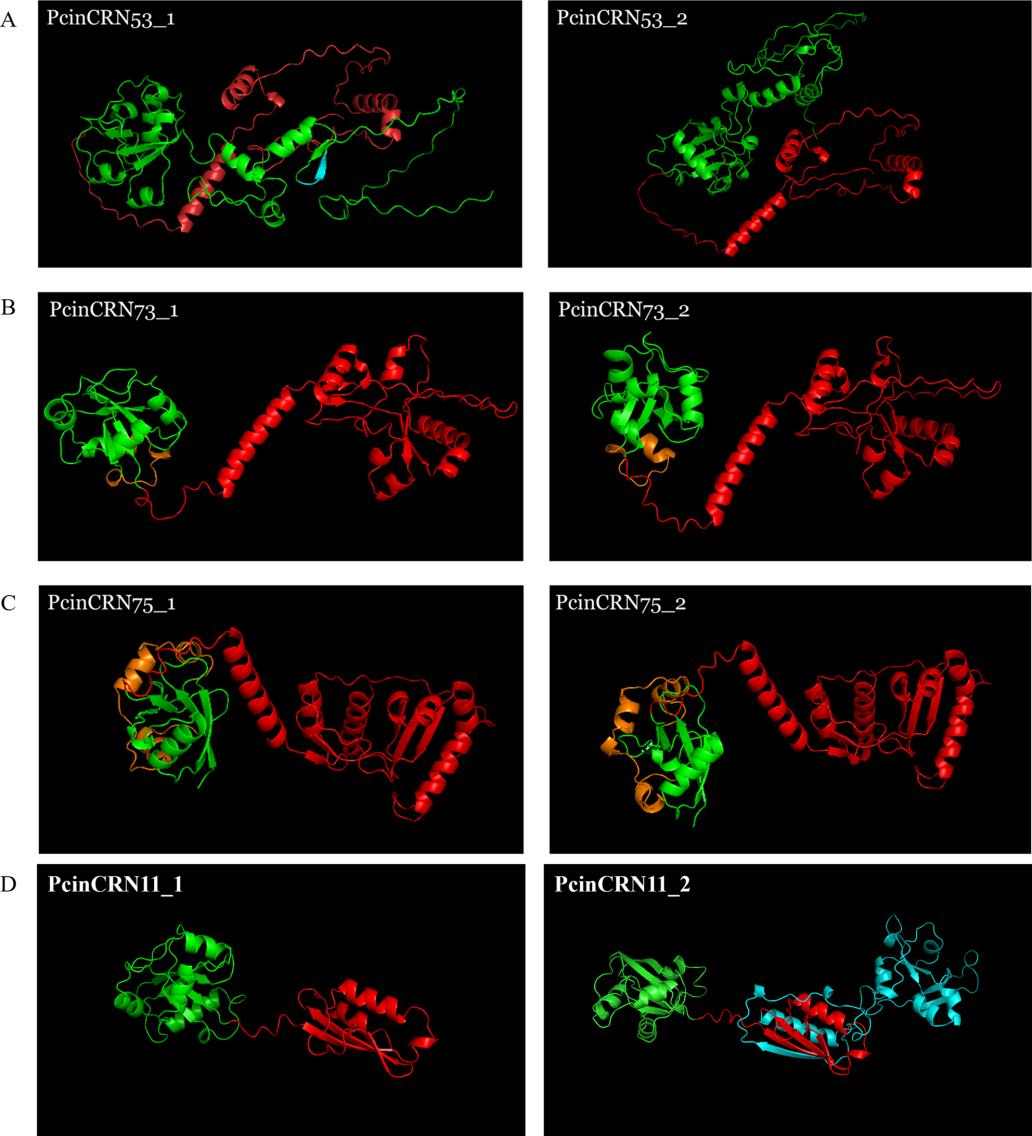
Predicted tertiary protein structures of the amino acid sequences encoded by the different PcinCRN alleles using AlphaFold. AlphaFold [29, 30] was used to predict the tertiary structure of *Phytophthora cinnamomi* crinkling and necrosis (PcinCRN) effector protein alleles. The predicted tertiary structures were visualised in PyMOL v2.5.5 (Schrödinger, LLC). (**A**) Tertiary protein structures of PcinCRN53_1 (pLDDT = 68.76) and PcinCRN53_2. (pLDDT = 67.05). The portion of the protein structure represented by a blue colour indicates the amino acids that are present in PcinCRN53_1 and not PcinCRN53_2. (**B**) Tertiary protein structure of PcinCRN73_1 (pLDDT = 81.70) and PcinCRN73_2 (pLDDT = 81.42). (**C**) Tertiary protein structure of PcinCRN75_1 (pLDDT = 89.02) and PcinCRN75_2 (pLDDT = 97.97). (**C**) Tertiary protein structure of PcinCRN11_2 (pLDDT = 84.52) and PcinCRN11_1 (pLDDT = 80.17). Portions of the protein structure represented by a green colour indicate the N-terminal domain up until the HVLVXXP motif. Orange coloured protein structures represent regions of amino acid variation within the N-terminal which differ between the alleles of PcinCRN73 and PcinCRN75. Portions of the proteins represented by a red colour indicate the location of the C-terminal domains. Blue labelled structure represents the additional protein structure that resulted due to the inclusion of an additional exon in PcinCRN11_2 compared to PcinCRN11_1, due to alternative splicing

The protein structures of PcinCRN11, PcinCRN53, PcinCRN73, PcinCRN75 and PcinCRN95 were predicted and compared. The amino acid changes resulting from SNPs, consecutive base substitutions, and deletions in the alleles of PcinCRN53, PcinCRN73 and PcinCRN75 impacted the structure of the protein (Fig. 9). PcinCRN73 and PcinCRN75 allele structural variations were present in the N-terminal of the protein, rather than the functional C-terminal (Fig. 9B and C). PcinCRN53 demonstrated an orientation shift based on the non-synonymous amino acid changes and deletions between alleles (Fig. 9A). Comparison of the predicted protein structure of PcinCRN95_1 and PcinCRN95_2 showed no notable structural differences between them (Supplementary Fig. 4). The predicted amino acid PcinCRN11_1 is alternatively spliced to produce PcinCRN11_2, allowing for an additional protein structure in the final tertiary structure (Fig. 9C).

## Discussion

*Phytophthora* effectors are known to play a role in cell death during infection of host plants by either inducing or suppressing cell death [13, 18–20, 23, 24, 27, 31–33]. Effectors such as CRNs may be utilized by *Phytophthora* spp. to manipulate the cell death pathways of the infected host plant to maintain their biotrophic and necrotrophic lifestyles, at different stages of infection [3, 13–15, 18, 19]. Currently, there is a lack of functional characterization studies on PcinCRNs. Two previous studies identified putative PcinCRN effector repertoires [1, 26], and another study revealed that a single *PcinCRN* (*CRN1*) was highly expressed during infection of *E. nitens* [25]. To that end, the current study identified a repertoire of 25 full-length PcinCRNs and one partial or CRN-like sequence. Putative cell death manipulating functions were assigned to a subset of PcinCRNs based on their expression during infection of *P. americana*, Sanger Sequencing data, relatedness to other *Phytophthora* CRNs functioning in cell death manipulation, protein domain analyses, and their tertiary protein structure.

Initial work by Hardham and Blackman [1] identified 49 putative PcinCRNs due to their similarity to CRNs from other *Phytophthora* spp. and the presence of a LXLFLAK and DWL motif (Fig. 1). Although many PcinCRNs were identified at the time, seven were incomplete. The two *P. cinnamomi* genomes used for the Hardham and Blackman [1] study were sequenced using Illumina Hi-Seq 2500 platform resulting in highly fragmented assemblies [34, 35]. The most recent identification of PcinCRNs was performed by Engelbrecht et al. [26], where 49 putative PcinCRNs were identified – of which two were truncated. In their study, a new *P. cinnamomi* reference genome was generated using a combination of Nanopore and Illumina sequencing platforms. This approach resulted in a less fragmented genome, with 133 scaffolds vs. 1314 and 10,084 scaffolds, N50 of 1.18 Mb compared to 10 and 264.5 Kb, and estimated genome size of 109.7 Mb compared to 53.69 and 77.97 Mb.

With this, the same *P. cinnamomi* genome from Engelbrecht et al. [26] was used in the current study to search for putative PcinCRN effectors via a HMM profile search, in order to generate the most accurate representation of the *P. cinnamomi* CRN repertoire possible. Although this approach resulted in less putative PcinCRNs being identified compared to Hardham and Blackman [1], and Engelbrecht et al. [26], the number of validated ‘true’ PcinCRNs from the current repertoire was greater (Fig. 2, Supplementary Table 3A and 3B). All ‘true’ PcinCRNs identified by Engelbrecht et al. [26], and eight of those from Hardham and Blackman [1] were among the list of ‘true’ PcinCRNs generated using the current studies method (Supplementary Table 3A and 3B). It was also found that two putative PcinCRNs from Hardham and Blackman [1] were not among our list of putative PcinCRNs, or that of Engelbrecht, et al. [26]. . The most likely reason for this would be an assembly-based artifact resulting from the highly fragmented genomes used in that study. Thus, based on our results we are confident that we have compiled a list of PcinCRNs which most accurately represents the true *P. cinnamomi* CRN effector repertoire.

We further assigned putative functions in cell death manipulation to PcinCRNs through multiple lines of evidence (Table 3). This included analyzing *PcinCRN* expression during *P. americana* infection (Fig. 3). Timepoints chosen to represent the biotrophic stage were 6- and 12 hpi, and the timepoint chosen to represent the necrotrophic stage was at 120 hpi, based on previous findings [36] (Fig. 1). Based on the study by van den Berg et al. [35]. the 24 hpi was used to represent the point at which *P. cinnamomi* was most likely transitioning from a biotrophic to a necrotrophic lifestyle [36]. Expression profiles, in combination with sequencing data of PcinCRN and their alleles, relatedness to other functionally characterized *Phytophthora* CRNs and the tertiary protein predictions were used to assign function. Ultimately 10 full-length PcinCRNs were functionally characterized as either a cell death inducer, suppressors, or as having contradictory function in cell death manipulation.

**Table 3:** Summary of evidence supporting the assignment of putative function in cell death manipulation to 10 full-length *Phytophthora cinnamomi* crinkling and necrosis (PcinCRN) effectors. The results obtained from expression profiles, Sanger Sequencing data, phylogenetic analyses, and protein analyses for each PcinCRN were accumulated as evidence towards the classification of the PcinCRNs as either a cell death suppressor or inducer. PcinCRN alleles were classified as performing contradicting functions or functions in alternative host plant species if the evidence supports the classification of the PcinCRN as a cell death inducer and suppressor. PcinCRNs were classified as potentially playing a role in the incompatible plant-pathogen interaction if they had statistically differentiated expression during infection of R0.12 compared to Dusa®

PcinCRN ID	Expression profile	Sanger Sequencing results	Phylogenetic relatedness	Protein analyses	Putative function
PcinCRN11	Upregulated during biotrophic stage and potential switch between lifestyles in an incompatible plant-pathogen interaction vs. compatible (Fig. 3C and E)	Alternative splicing occurs in *PcinCRN11_1* resulting in a *PcinCRN11_2* variant (Table 2; Fig. 6)	All variants form a clade with a known cell death inducer (PiCRN1) (Fig. 7)	Contains both Central & Terminal LCRs (Table 1)	Cell death Inducer/suppressor
	Downregulated during initial infection in R0.12 (Fig. 3C)	No alleles			Variants may perform contradicting functions
					Potentially plays a role in the incompatible interaction between avocado and *P. cinnamomi*
PcinCRN30	Upregulated during the biotrophic stage in Dusa® (Fig. 3B and D)	Two alleles with 1 amino acid difference. (Table 2, Supplementary Fig. 3B)	Both alleles were similar to a known cell death suppressor (PsCRN108) (Fig. 7)	Contains Central LCR. (Table 1)	Cell death suppressor
PcinCRN52	Upregulated during necrotrophic phase in R0.12 (Fig. 3A)	No alleles	Similarity to a CRN known to induce cell death (PcCRN4) (Fig. 7)	Contains a Central LCR & NLS. (Table 1)	Cell death inducer
PcinCRN53	Upregulated during biotrophic stage or potential switch between lifestyles in an incompatible plant-pathogen interaction vs. compatible (Fig. 3C and E)	Two alleles with seven amino acid differences and deletion of four amino acids (Table 2; Fig. 4)	Forms a clade with a known cell death inducer (PiCRN1) (Fig. 7)	Demonstrates an orientation shift based on the amino acid changes and deletions between alleles which will change the function between alleles (Fig. 9A)	Cell death Inducer/suppressor
					Alleles may perform contradicting functions or functions in alternative host plant species
	Downregulated during initial stage of infection compared to the necrotrophic phase in Dusa® (Fig. 5)			Both alleles Contain NLS (Table 2)	Potentially plays a role in the incompatible interaction between plant and pathogen
PcinCRN73	Upregulated during biotrophic phase and downregulated during necrotrophic phase in R0.12 (Fig. 3A and C)	Two alleles with seven amino acid differences and deletion of two amino acids in PcinCRN73_1 and five deletions in PcinCRN73_2 (Table 2; Fig. 5)	Forms a clade with a known cell death inducer (PiCRN1) (Fig. 7)	Protein structure variations between alleles are within the N-terminal of the protein. (Fig. 9B)	Cell death suppressor/inducer
	Downregulated during initial infection at 6 hpi in R0.12 (Fig. 4)				Alleles may perform contradicting functions or functions in alternative host plant species
PcinCRN75	Upregulated during biotrophic stage or potential switch between lifestyles in an incompatible plant-pathogen interaction vs. compatible (Fig. 3C and E)	Two alleles with 34 amino acid differences and deletion of three amino acids (Table 2; Fig. 5)	Forms a clade with a known cell death inducer (PiCRN1) (Fig. 7)	Protein structure variations between alleles are within the N-terminal of the protein. (Fig. 9C)	Cell death suppressor/inducer
					Alleles may perform contradicting functions or functions in alternative host plant species
	Upregulated during initial stage of infection compared to the necrotrophic phase in Dusa® (Fig. 3D)				Potentially plays a role in the incompatible interaction between plant and pathogen
PcinCRN77	Upregulated during biotrophic stage and potential switch between lifestyles in an incompatible plant-pathogen interaction vs. compatible (Fig. 3E)	No alleles (Table 2)	Forms a clade with a known cell death inducer (PiCRN1) (Fig. 7)	N/A	Cell death suppressor
					Potentially plays a role in the incompatible interaction between plant and pathogen
PcinCRN81	Upregulated during the biotrophic stage in Dusa® (Fig. 3B)	Two alleles with one amino acid difference (Table 2, Supplementary Fig. 3)	Both alleles were similar to a known cell death suppressor (PsCRN108) (Fig. 7)	Contains both Central & Terminal LCRs (Table 1)	Cell death suppressor
PcinCRN86	Downregulated during necrotrophic stage of R0.12 and Dusa® (Fig. 3A and B)	No alleles	N/A	Contains Terminal LCR (Table 1)	Cell death suppressor
	Upregulated during the biotrophic stage in Dusa® (Fig. 3D)				
PcinCRN95	Upregulated during the biotrophic phase in Dusa® (Fig. 3B and D)	Two alleles with seven amino acid differences (Table 2; Fig. 4)	Both alleles were similar to a CRNs known to induce cell death (PiCRN5, PiCRN8) (Fig. 7)	Contains Central LCR (Table 1)	Cell death inducer/suppressor
				Contains Ubl and P-loop NTPase domain – like redefined architecture of CRNs involved in cell death (Fig. 9D)	Alleles may perform contradicting functions or functions in alternative host plant species
	Downregulated during necrotrophic phase of Dusa® (Fig. 3B)				
	Upregulated during necrotrophic stage and potential switch between lifestyles in an incompatible plant-pathogen interaction vs. compatible (Fig. 3E)				

Previous research by Meyer et al. [25]. clearly demonstrated that upregulation of *P. cinnamomi CRN1* during the late-stages of infection (120 hpi) in *E. nitens* was associated with virulence. Additionally, overexpression of *PcCRN4* in *N. benthamiana* led to induction of cell death [19]. Our data demonstrated that *PcinCRN52* was significantly upregulated at 120 hpi, the timepoint considered to represent the necrotrophic phase of infection during the incompatible *P. cinnamomi-P. americana* interaction [36, 37], similar to that of *CRN1* from Meyer et al. [25]. (Fig. 3A; Table 3). PcinCRN52 was also found to be related to PcCRN4 (Fig. 7; Table 3). Notably, like PcCRN4, PcinCRN52 contains a central LCR which is essential in manipulation of host translation and transcription processes [38, 39]. Additionally, PcinCRN52 contained a NLS (Tables 2 and 3) which is known to assist in the nuclear localisation of several CRNs [13, 18, 19, 21, 23]; in fact, most identified cell death inducing CRNs localise to the nucleus through a NLS or alternative mechanisms, including PcCRN4 [13]. This domain allows for nuclear localisation of CRNs where they enact their function and regulate the expression of cell death-related genes [11, 12, 18]. Thus, our temporal expression data, structural and phylogenetic analyses would suggest that PcinCRN52 may function as a cell death inducer.

By contrast, four PcinCRNs (*PcinCRN30, PcinCRN77*, *PcinCRN81* and *PcinCRN86*) were determined to play a potential role in suppressing cell death during the biotrophic stage of infection of avocado. Notably, these PcinCRNs displayed upregulation in expression during the biotrophic stage followed by downregulation during the necrotrophic stage (Fig. 3B-D; Table 3) [40]. PsCRN115 is known to suppress cell death induced by other cell death inducing *Phytophthora* effectors, and this CRN is upregulated at 12 hpi (biotrophic stage) during infection of *Glycine max* (soybean) compared to later time points [18]. PcinCRN30 and PcinCRN81 were found to be related to PsCRN108, a CRN known to suppress cell death during infection [32, 33]. Although PcinCRN77 was found to be related to a PiCRN1 [20], a cell death inducer, the expression data of our study provides stronger evidence to the designation of this CRN as a cell death suppressor during infection of avocado (Fig. 3C). Additionally, *PcinCRN30, PcinCRN77*, *PcinCRN81* and *PcinCRN86* did not contain a predicted NLS (Table 2). This however is not evidence that these PcinCRNs do not localize to the nucleus, as there are various alternative methods and/or pathways for translocation into the nucleus [27, 41]. Although, it is expected that these PcinCRNs do not localize to the nucleus because CRNs functioning in cell death suppression often act within the cytosol [11, 41]. This is because the primary targets of pathogen-associated molecular pattern (PAMP) triggered immune response (PTI) and the effector-triggered immune response (ETI) are found in the cytosol [42–44]. The PTI and ETI systems influence host-pathogen interactions and involve the activation of complex signaling pathways through a repertoire of proteins in response to pathogen attack. PcinCRN30, PcinCRN77, PcinCRN81, and PcinCRN86 potentially suppress cell death by targeting PTI and ETI related proteins to prevent a mounted immune response by the host plant.

Sequencing data revealed that multiple *PcinCRN* genes (*PcinCRN53, PcinCRN73, PcinCRN75*, and *PcinCRN95*) have two alleles with more than one amino acid difference between them (Table 2; Figs. 4 and 5). All these *PcinCRNs* exhibited the expression profile of a cell death suppressor (Fig. 3A, C- E; Table 3) but PcinCRN53, PcinCRN73 and PcinCRN75 were found to be phylogenetically related to PiCRN1, and PcinCRN95 to both PiCRN5 and PiCRN8, all of which are cell death inducers from *P. infestans* (Fig. 7; Table 3). Additionally, the PcinCRN95 protein architecture was indicative of a cell death inducer (Fig. 8). By containing a Ubl and P-loop NTPase domain (Fig. 8), PcinCRN95 is similar to the architecture of cell death-inducing CRNs defined by Zhang et al. [28]. The authors reported that majority of the cell death inducing CRNs from *P. infestans* and *P. sojae* possessed a Ubl domain in the N-terminal, followed by a NTPase and Restriction endonuclease (REase) domain in the C-terminal (Fig. 8). PcinCRN95 was found to be closely related to PiCRN8 (Fig. 7), a *P. infestans* CRN known to contain a REase4 domain [24]. No REase domain was predicted for PcinCRN95, but this may simply be due to the notable sequence diversity among REase domains and a lack of characterised CRNs [24, 28]. . We hypothesize that the contradicting nature of evidence, as well as the presence of alleles for *PcinCRN53, PcinCRN73, PcinCRN75*, and *PcinCRN95*, is because one allele encodes for a cell death inducer and the other allele encodes a protein functioning in cell death suppression.

To illustrate that PcinCRNs with two alleles may encode proteins with contradictory function in cell death, the tertiary proteins for these alleles were predicted (Fig. 9; Table 3). The predicted protein structure of PcinCRN95_1 and PcinCRN95_2 revealed no difference in protein folding due to the amino acid changes (Supplementary Fig. 4). However, this does not imply that they lack contradictory functions in cell death. For example, PsCRN63 and PsCRN115 from *P. sojae* only differ by four amino acids and they perform contradicting functions in cell death [11, 18]. Investigations uncovered that PsCRN63 induces cell death and requires nuclear localization to function. Whereas, PsCRN115 functioned in cell death suppression during the necrotrophic stage and did not require nuclear localization to function [18]. Additionally, it was determined that PsCRN115 was able to suppress the cell death induced by PsCRN63, and that silencing one or both genes had negatively impacted virulence. This mechanism has also been observed in *Phytophthora parasitica*, where PpCRN7 and PpCRN20 function the same as PsCRN63 and PsCRN115, respectively [12]. The interaction and manipulation observed between the two CRNs in *P. sojae* and *P. parasitica* may resemble the interaction and function of PcinCRN95_1 and PcinCRN95_2 during infection. Moreover, the tertiary protein structures between PcinCRN53_1 and PcinCRN53_2 were altered – where there is a structure deleted in the N-terminal and the orientation of the functional C-terminal is shifted (Fig. 9A). Due to these changes, the different PcinCRN53 proteins could potentially play contradicting roles in cell death manipulation like that of PcinCRN95. Conversely, the changes in the folding of the tertiary protein structures potentially allow for their functionality in different host plant species or their binding to different host plant targets. This may explain why *P. cinnamomi* (∼ 5000 host plants worldwide) has a larger host range than other *Phytophthora* spp [7, 8]. . This is evident when looking at the protein predictions for the proteins encoded by different alleles of PcinCRN73 and PcinCRN75 (Fig. 9B and C). The amino acid changes resulting between the alleles mainly occur in the N-terminal, rather than the C-terminal, indicating the changes may alter binding of the PcinCRNs to host targets [21, 45].

Confirmation of the coding sequence of *PcinCRNs* not only revealed the presence of alleles for some *PcinCRNs*, but one *PcinCRN* was demonstrated to undergo alternative splicing (*PcinCRN11*) (Table 3; Fig. 6). The *PcinCRN11* gene is alternatively spliced to produce variants *PcinCRN11_1* and *PcinCRN11_2*, where PcinCRN11_2 has an additional protein structure compared to PcinCRN11_1 (Fig. 9D; Table 3). This is the first evidence of a *Phytophthora* CRN gene undergoing alternative splicing. The expression of this *PcinCRN* was found to be upregulated in the susceptible rootstock (R0.12) when compared to the partially resistant rootstock (Dusa®) during the biotrophic stage, indicating this PcinCRN may serve a role in the susceptibility of host plants to *P. cinnamomi.* However, it was shown that PcinCRN11 forms a clade with a cell death inducing *P. infestans* CRN (PiCRN1), and that Like other PcinCRNs, these variants of PcinCRN11 could potentially perform contradicting functions in cell death manipulation, but whether PcinCRN11_2 functions as a cell death suppressor or inducer will have to be determined.

In addition to *PcinCRN11*, other PcinCRNs were suggested to contribute to the susceptibility during a *P. cinnamomi* – *P. americana* incompatible interaction. A previous study was conducted by *Li* et al. [46] where the global expression profiles during a compatible and incompatible *P. infestans* - *Solanum tuberosum* interaction was investigated using dual RNA-seq. A total of five *PiCRN* genes were found to be expressed at 24 hpi of an incompatible interaction that were not detected in the compatible interaction. Similarly, *PcinCRN11*, *PcinCRN53, PcinCRN73* and *PcinCRN75* were found to be upregulated during an incompatible interaction (R0.12) compared to the compatible interaction (Dusa®) at either or both 12- and 24 hpi (Fig. 3E; Table 3). A partially resistant rootstock is defined by having minor symptoms due to a decreased pathogen load, and the HR is a plant defense response to inhibit the spread of a pathogen [15, 16]. Due to *P. cinnamomi* being a hemi-biotroph, the HR would benefit the host plant during the biotrophic stage of the pathogen. Therefore, the increased expression of PcinCRNs associated with cell death suppression during the biotrophic stage of a susceptible rootstock compared to the partially resistant rootstock was expected since these PcinCRNs serve in suppressing the HR, ultimately aiding in the spread of the pathogen. Alternatively, PcinCRN95 was found to be upregulated during an incompatible interaction (R0.12) compared to a compatible interaction (Dusa®) at 120 hpi (Fig. 3E; Table 3). We suggest that PcinCRN11, PcinCRN53, *PcinCRN73, PcinCRN75* and *PcinCRN95* play a role in the susceptible outcome during a *P. cinnamomi* – *P. americana* incompatible interaction.

## Conclusion

With CRN effector proteins playing a potential role in manipulating cell death during the biotrophic and necrotrophic stages of infection by *P. cinnamomi*, the identification and characterization of these effectors are crucial to our understanding of the infection and colonization tactics employed by *Phytophthora* spp. We provide an up-to-date representation of the *P. cinnamomi* CRN effector protein repertoire and are the first to sequence and assign putative function in cell death manipulation to 10 PcinCRNs. With the availability of the full coding sequences of PcinCRNs and their variants, future functional characterization studies in *P. cinnamomi* can be done. With the availability of methods such as Agroinfiltration and CRISPR-Cas knockout, the functions of the identified PcinCRN presented in this paper can be confirmed and their roles in virulence determined. This will contribute to our knowledge of *P. cinnamomi* cell death pathways and their host targets, allowing for improved screening of resistant avocado rootstocks to be used in agricultural practices.

### Methods

#### Identification of full-length PcinCRN effector protein sequences

**Fig. 10:**
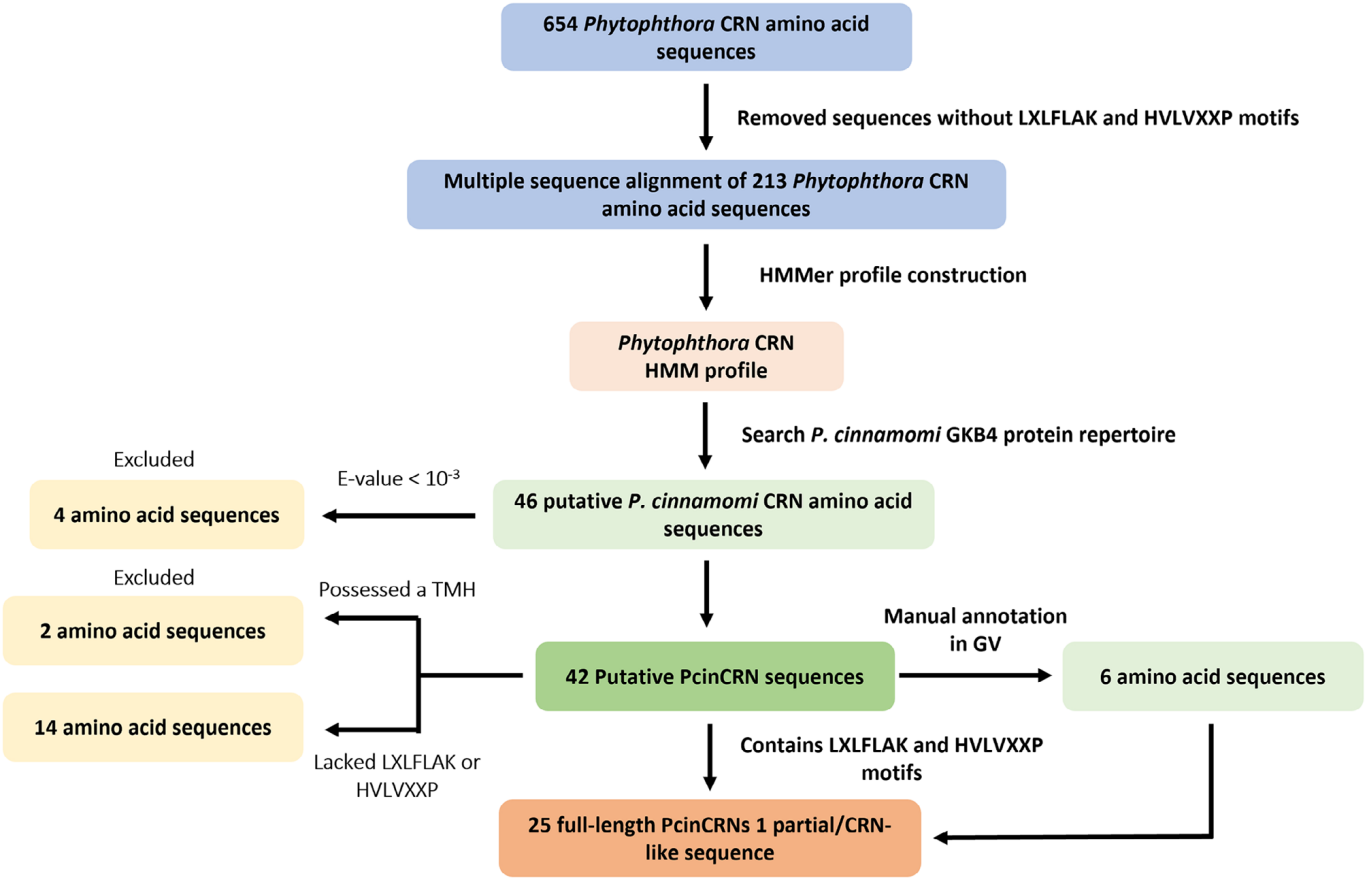
Schematic depicting PcinCRN identification and validation pipeline. Workflow to create a *Phytophthora* crinkling and necrosis (CRN) hidden Markov model (HMM) profile and exclusion criteria that resulted in the final repertoire of ‘true’ *Phytophthora cinnamomi* CRN (PcinCRN) effector proteins. A total of 213 *Phytophthora* CRN amino acid sequences were validated as ‘true CRNs from a list of 654 putative *Phytophthora* CRNs, and used to generate a multiple sequence alignment which was subsequently used to construct a *Phytophthora* CRN HMM profile. The HMM profile was used to search the *P. cinnamomi* GKB4 genome, resulting in the identification of 46 putative PcinCRN amino acid sequences. Four putative PcinCRNs were excluded because of an E-value < 10^− 3^. An additional 16 putative PcinCRN sequences were excluded because they lacked one or both conserved motifs (LXLFLAK and HVLVXXP) or had a transmembrane helix (TMH). A final list of 25 full-length sequences were confirmed as ‘true’ *Phytophthora* CRNs and a partial/CRN-like sequence. Six of the 25 PcinCRNs were manually annotated in GV

A pipeline was generated to identify and validate PcinCRNs as ‘true’ *Phytophthora* CRNs from the *P. cinnamomi* GKB4 genome (Fig. 10). *Phytophthora* CRN protein sequences obtained from the NCBI database were validated by confirming the presence of the LXLFLAK and HVLVXXP motifs in the N-terminus (Supplementary Table 12), using QIAGEN CLC Main Workbench v8.0 (https://digitalinsights.qiagen.com/), and then used to generate a multiple sequence alignment. To identify putative *P. cinnamomi* CRN (PcinCRN) protein homologs, a HMM profile was generated in HMMER v3.3.2 (http://hmmer.org/) using the multiple sequence alignment to search the full *P. cinnamomi* GKB4 protein repertoire predicted by Augustus [26]. Homologous PcinCRN protein sequences identified with an E-value > 10^− 3^ were excluded. Putative PcinCRN protein sequences were analysed for the presence of both the LXLFLAK and HVLVXXP conserved motifs in the N-terminal using CLC Main Workbench, allowing for a single amino acid difference in each motif. Putative PcinCRN sequences lacking either or both conserved motifs were excluded. The presence of TMH within the putative protein sequences was determined using TMHMM v2.0 (Technical University of Denmark) (https://services.healthtech.dtu.dk/services/TMHMM-2.0/) [47] with default parameters. Putative sequences containing a TMH were excluded. SignalPv3.0 was used to predict the presence of a signal peptide in the remaining candidate sequences. Domains within the PcinCRN protein sequences were identified using SMART (http://smart.embl-heidelberg.de/) [48]. A putative CRN sequence was considered a full-length PcinCRN if the encoded amino acid sequence contained both the LXLFLAK and HVLVXXP motifs and lacked a TMH. The final list of PcinCRN protein sequences were cross-referenced against a suite of putative PcinCRN protein sequences identified in two previous studies, where the BLAST2GO method was used [1, 26]. PcinCRN protein sequences from Hardham and Blackman [1], and Engelbrecht et al. [26] were analysed using the same method described above. In the case where the predicted protein sequences of the putative PcinCRNs were missing a HVLVXXP conserved motif, the protein sequences were manually annotated in GenomeView 2250 (GV) [49]. Of the sequences that were missing the HVLVXXP motif, the last intron was analysed to determine if the exon-intron boundaries were incorrectly predicted and if the HVLVXXP motif was present downstream of the original exon prediction. If the HVLVXXP motif was absent, the sequence was discarded.

### Analysis of *P. Cinnamomi CRN* expression profiles

The expression data of 25 *PcinCRN* effectors were obtained by previously generated dual RNA-seq data of susceptible (R0.12) and partially resistant (Dusa®) *P. americana* rootstocks inoculated with *P. cinnamomi* GKB4 [50]. Briefly, RNA-seq reads were trimmed, and low-quality bases were removed using Trimmomatic v. 0.39 [51]. The read quality was confirmed using FASTQC v. 0.11.9, after which the reports were summarized using MultiQC [52]. RNA-seq reads were aligned to the *P. cinnamomi* genome using HISAT v. 2.0.6 [53]. Transcript abundance was quantified within RNA-seq libraries across all time-points (6-, 12-, 24- and 120 hpi) using featureCounts v. 2.0.1 [54], where *P. cinnamomi* mycelia was used as a reference library. The normalization and analysis of counts were performed using DESeq2 [55]. The Wald test was used to obtain data for differentially expressed genes (DEGs) at each time-point, and statistical significance was assigned using the Benjamini-Hochberg false discovery rate (FDR) method. Significant DEGs were identified as those with a Log_2_ (Fold Change) ≥ 1 or ≤-1 while the statistical significance of the observations were determined using an FDR cut-off (adjusted *p*-value) of ≤ 0.05 and ≤ 0.10. Expression data for candidate *PcinCRN* genes were extracted from the output of DESeq2 using a custom R script [56]. Pheatmap version 1.0.12 was used to generate heatmaps for expression data visualization [57]. The expression of each *PcinCRN* was analysed by first comparing the expression of candidate *PcinCRN* genes at 6-, 12-, 24- and 120 hpi - in both the susceptible and partially resistant rootstocks - to mycelia, and then by comparing the expression of candidate *PcinCRN* in susceptible rootstock to the expression in the partially resistant rootstock. Data comparing the expression between different time-points within each rootstock were also obtained.

### Validation of *PcinCRN* expression using RT-qPCR

Reverse transcriptase (RT)- quantitative (q)PCR was used to validate the expression of four *PcinCRN* genes (*PcinCRN74, PcinCRN79, PcinCRN90*, and *PcinCRN95*). Using PrimerQuest™, primers for target *PcinCRNs* were designed (Integrated DNA Technologies, Coralville, USA). Primer sequences for candidate endogenous control genes ubiquitin-conjugated enzyme (*Ubc*), Beta-tubulin (*β-tubulin*), and *WS041* were obtained from literature [58, 59] (Supplementary Table 13 A). By generating standard curves with five-fold dilutions of a *P. cinnamomi* cDNA pool, the efficiency of the respective primers was determined (Supplementary Figs. 5 and 6). In the RT-qPCR reaction, 200 ng of previously prepared cDNA during *P. cinnamomi* GKB4 infection of a susceptible *P. americana* rootstock (R0.12) at 12- and 24 hpi served as the template in RT-qPCR expression analysis, while mycelia served as the control. For the RT-qPCR reactions, three biological replicates representing each time-point (12- and 24 hpi) as well as three mycelial control samples were utilized for each target and reference gene. The RT-qPCR experiment was conducted using the KAPA SYBR® FAST qPCR Master Mix (2X) Kit (Roche, Mannheim, Germany) according to the manufacturer’s instructions on the BioRad CFX96 Touch™ Real-Time PCR Detection System (Bio-Rad Laboratories Inc, Hercules, United States of America (USA)). For each target and reference gene, melt curves were generated and analysed using CFX Maestro™ 1.1 (Bio-Rad Laboratories Inc). The software package qbase + 3.2 (Biogazelle, Zwijnaarde, Belgium) was utilized for normalization and relative quantification. Microsoft® Excel 2016 was used to calculate the Log_2_ (Fold Change) for each target gene using the method described by Pfaffl (2001) [60]. Microsoft® Office Excel 2016 was used to conduct a two-tailed t-test to determine statistical significance.

### Amplification of *PcinCRN* coding sequences from *P. Cinnamomi* cDNA

Primers were designed using PrimerQuest™ for amplification of the candidate *PcinCRN* coding sequences from *P. cinnamomi* cDNA - previously synthesised from RNA isolated during *P. cinnamomi* GKB4 infection of a susceptible *P. americana* rootstock (R0.12) at 6-, 12-, 24 hpi. Primers were designed to bind within the upstream and downstream untranslated regions and within the predicted coding sequences of each *PcinCRN* (Supplementary Table 13B). *PcinCRNs* were amplified from cDNA by PCR using Phusion Green Hot Start II High-Fidelity DNA Polymerase (Thermo Fisher Scientific, Waltham, USA). Reagent concentrations for reactions: 1X Phusion HF buffer, 200 µM dNTPs, 0.2 U Phusion Green Hot Start II High-Fidelity DNA Polymerase, 100 ng *P. cinnamomi* GKB4 cDNA and 0.5 µM of each primer. A Veriti 96 Well Thermal Cycler (Thermo Fisher Scientific) was used: initial denaturation at 98 °C for 1 min, 25 cycles of 10 s denaturation at 98 °C, annealing stage was omitted due to the Tm being > 69 °C (except for *PcinCRN95* fragment A1, where 30 s annealing at 66 °C was used) and 30 s extension at 72 °C, and a final extension for 10 min at 72 °C. PCR products were excised from a 2% agarose gel and purified using Zymoclean™ Gel DNA Recovery Kit (Zymo Research, USA) according to manufacturer’s instructions. The concentration of each purified amplicon was determined using a NanoDrop™ 2000 Spectrophotometer (Thermo Fisher Scientific).

### Cloning and sequencing of *PcinCRN* coding sequences

The *PcinCRN* amplicons were cloned using the Zero Blunt® TOPO® PCR Cloning Kit (Thermo Fisher Scientific). The cloning reaction was prepared based on the manufacturer’s guidelines, where 15–30 ng of *PcinCRN* PCR product was used. The full volume of the cloning reaction was transformed into *Escherichia coli* DH5α competent cells using chemical transformation. Transformed cells were plated on LB/Kan50 agar plates (2.5% w/v LB medium, 1.5% w/v agar bacteriological, 0.1% v/v 50 µg/ml Kanamycin) and incubated overnight at 37 °C. Three transformants for each *PcinCRN* amplicon were selected for plasmid extraction. Transformants were inoculated into 5 ml LB/Kan50 broth and incubated overnight at 37 °C with shaking (150 rpm). Plasmids were extracted using QIAprep® Spin Miniprep Kit (QIAGEN, Hilden, Germany), with the following modifications to the manufacturer’s protocol: 4 ml of overnight culture was collected by centrifugation at 13, 000 rpm for 1 min at room temperature; the PB buffer wash step was added; 30 µl of EB buffer was used to elute DNA and allowed to stand for 5 min prior to centrifugation. The concentration of the plasmid extractions was determined using a NanoDrop™ 2000 Spectrophotometer. The plasmid extractions were sequenced via Sanger sequencing using BigDye® Terminator v3.1 Cycle Sequencing Kit (Thermo Fisher Scientific) and vector specific M13 primers (Supplementary Table 13B). Each *PcinCRN* was sequenced in both the forward and the reverse orientation. Each sequencing reaction contained: 0.85 X Sequencing buffer, 4.17% v/v BigDye 3.1, 0.83 µM primer, 40–200 ng plasmid DNA. The sequencing reaction was done in the Veriti 96 Well Thermal Cycler, set for an initial denaturation at 96 °C for 5 s, followed by 25 cycles of 10 s denaturation at 96 °C, 5 s annealing at 55 °C and 4 min extension at 60 °C.

The sequencing products were precipitated using a sodium acetate protocol, as follows: 60 µl of a precipitation mixture containing 2 µl NaOAc 3 M, pH 5.2, and 50 µl 100% ethanol was added to each sequencing product. The tubes were incubated on ice for 15 min and centrifuged at 12, 000 g for 30 min. Ethanol (70% v/v) was used to clean the DNA pellet twice, each followed by centrifugation at 12, 000 g for 10 min. The supernatant was removed, and the DNA pellets were dried in a heating block set at 66 ºC for 10 min. All samples were submitted to the DNA Sanger sequencing facility at the University of Pretoria for sequencing using an ABI 3500xl genetic analyser (Thermo Fisher Scientific). The presence of a NLS was determined by submitting the translated amino acid sequences of each PcinCRN through NLStradamus [61] using a 4 state HMM static model with a Posterior cut-off of 0.4.

### Confirming the presence of *PcinCRN* alleles in two additional *P. Cinnamomi* isolates

Genomic DNA was extracted from freeze-dried mycelia of two different *P. cinnamomi* isolates (Pcin_isolate129 and Pcin_isolate308) using CTAB extraction protocol [62]. Both isolates were sampled from *P. cinnamomi* infected *P. americana* trees located in different orchards in Tzaneen, Limpopo, South Africa. The same amplification, cloning, extraction, and sequencing protocol as mentioned above was used to confirm the presence of *PcinCRN73, PcinCRN75, PcinCRN53* and *PcinCRN95* alleles.

### Protein modelling of confirmed full-length PcinCRN allele amino acid sequences

AlphaFold [29, 30] was used to predict the protein structure of PcinCRN sequences shown to have more than one allele. On a scale from 0 to 100, AlphaFold generated a per-residue confidence metric: predicted local distance difference test (pLDDT). A high pLDDT score (> 80) indicates high confidence in the structure of the residue, whereas a low pLDDT score (< 50) may indicate that the residues are in intrinsically disordered protein regions. The protein structures generated were visualized using the PyMOL Molecular Graphics System, Version v.2.3.0 (Schrödinger, LLC). The protein structures for the different alleles were compared to one another to determine whether the amino acid changes resulted in protein folding differences.

### Phylogenetic analysis

The amino acid sequences of putative CRNs from other *Phytophthora* spp. were obtained from the UniprotKB database (Uniprot Consortium, 2014) (Supplementary Table 14) and the full-length PcinCRN proteins identified and validated in this study were used. All sequences were trimmed after the HVLVXXP motif so that only the N-terminal was used in the alignment. The CRN amino acid sequences were aligned using MUSCLE in CLC Main Workbench. The alignment was subjected to Bayesian inference analysis using MrBayes 3.2.7a. in Geneious Prime 2022.2.2 (Biomatters, New Zealand), using the Poisson substitution model and a CRN from *Pythium ultimum* as an outgroup. In the analysis, one million generations of the Markov chain Monte Carlo (MCMC) analysis were used, with trees being sampled at every 200th generation. Following the MCMC analysis, 10% of the trees were discarded as burn-in phase, with the remaining trees being used to calculate posterior probabilities. A second phylogenetic analysis was performed using the same criteria as above, except the full-length sequences of confirmed PcinCRN amino acid sequences were compared to the full-length amino acid sequences of only functionally characterized CRNs from other *Phytophthora* spp. (Supplementary Table 15).

### Electronic supplementary material

Below is the link to the electronic supplementary material.

Supplementary Material 1

Supplementary Material 2

Supplementary Material 3

Supplementary Material 4

Supplementary Material 5

Supplementary Material 6

Supplementary Material 7

Supplementary Material 8

Supplementary Material 9

Supplementary Material 10

Supplementary Material 11

Supplementary Material 12

Supplementary Material 13

Supplementary Material 14

Supplementary Material 15

Supplementary Material 16

Supplementary Material 17

## Data Availability

Data generated or analysed during this study are included in this published article and its supplementary information files. Sequences used in this study are available on Genbank (NCBI) accession numbers OR501732 - OR501777. All *P. cinnamomi* cultures are available in the ARP culture collection at the University of Pretoria, South Africa.

## Abbreviations

HR: Hypersensitive Response
PCD: Programmed Cell Death
PRR: Phytophthora root rot
CRN: Crinkling and necrosis effector (Crinkler)
PsCRN: *Phytophthora sojae* CRN
PcinCRN: *Phytophthora cinnamomi* CRN
PpCRN: *Phytophthora parasitica* CRN
PcCRN: *Phytophthora capsici* CRN
PiCRN: *Phytophthora infestans* CRN
PmCRN: *Phytophthora megakarya* CRN
PuCRN: *Pythium ultimum CRN*
HMM: Hidden Markov Model
RNA-seq: RNA-sequencing
LCR: Low complexity regions
Ubl: Ubiquitin-like domain
P-loop: Phosphate-loop
REase: Restriction endonuclease
NTPase: Nucleoside-triphosphatase
HTH: Helix-turn-helix domain
TMH: Transmembrane helix
SNP: Single nucleotide polymorphism
INDEL: Insertion or deletion
NLS: Nuclear localisation signal
PAMP: Pathogen associated molecular pattern
PTI: PAMP triggered immune response
ETI: Effector triggered immune response
HSP: Heat shock proteins
HSE: Heat shock elements
hpi: Hours post-inoculation
