# Supplementary material for: Identification of Phytophthora cinnamomi CRN effectors and their roles in manipulating cell death during Persea americana infection
Source: BMC Genomics. 2024 May 2;25:435. doi: 10.1186/s12864-024-10358-3 (PMC11064341; doi:10.1186/s12864-024-10358-3)
Supplement: Supplementary file 1 — Supplementary Material 1 [file 12864_2024_10358_MOESM1_ESM.docx]

**E =** 91.8% **R^2^ =** 0.985 **Slope =** -3.535

B

A


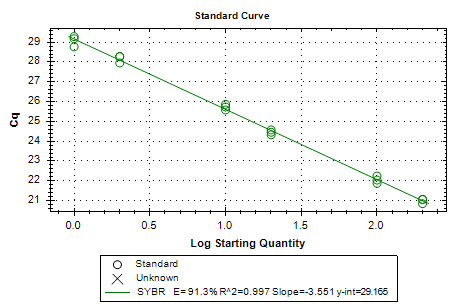

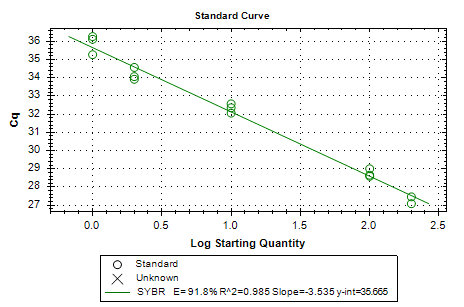


C

**E =** 91.3% **R^2^ =** 0.997 **Slope =** -3.551


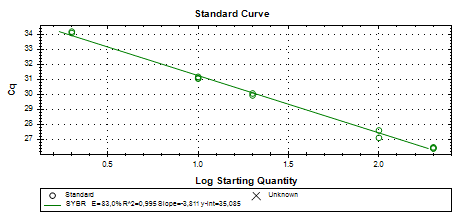


**E =** 83.0% **R^2^ =** 0.995 **Slope =** -3.811


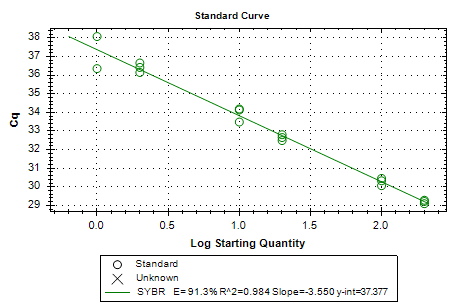


D

**E =** 91.3% **R^2^ =** 0.984 **Slope =** -3.550

**Supplementary Figure 6. Standard curves of target *PcinCRN* genes for RT-qPCR.** Standard curves of the reference genes were prepared from a 5-fold dilution series of a cDNA template pool. (A) *PcinCRN74*, (B) *PcinCRN79*, (C) *PcinCRN90* and (D) *PcinCRN95.* The log of the starting quantity was plotted against the Cq value for each of the standard wells and a line of best fit was drawn through the data points. The efficiency (E), slope and correlation (R^2^) values are indicated.
