# Supplementary material for: Identification of Phytophthora cinnamomi CRN effectors and their roles in manipulating cell death during Persea americana infection
Source: BMC Genomics. 2024 May 2;25:435. doi: 10.1186/s12864-024-10358-3 (PMC11064341; doi:10.1186/s12864-024-10358-3)
Supplement: Supplementary file 2 — Supplementary Material 2 [file 12864_2024_10358_MOESM2_ESM.docx]

**C**

**B**

**A**

**Supplementary Figure 5. Standard curves of RT-qPCR reference genes.** Standard curves of the reference genes were prepared from a 5-fold dilution series of a cDNA template pool. The (A) *Ubc*, (B) *B-Tubulin* and (C) *WS041.* The log of the starting quantity was plotted against the Cq value for each of the standard wells and a line of best fit was drawn through the data points. The efficiency (E), slope and correlation (R^2^) values are indicated.
