# Supplementary material for: Identification of Phytophthora cinnamomi CRN effectors and their roles in manipulating cell death during Persea americana infection
Source: BMC Genomics. 2024 May 2;25:435. doi: 10.1186/s12864-024-10358-3 (PMC11064341; doi:10.1186/s12864-024-10358-3)
Supplement: Supplementary file 4 — Supplementary Material 4 [file 12864_2024_10358_MOESM4_ESM.docx]

**
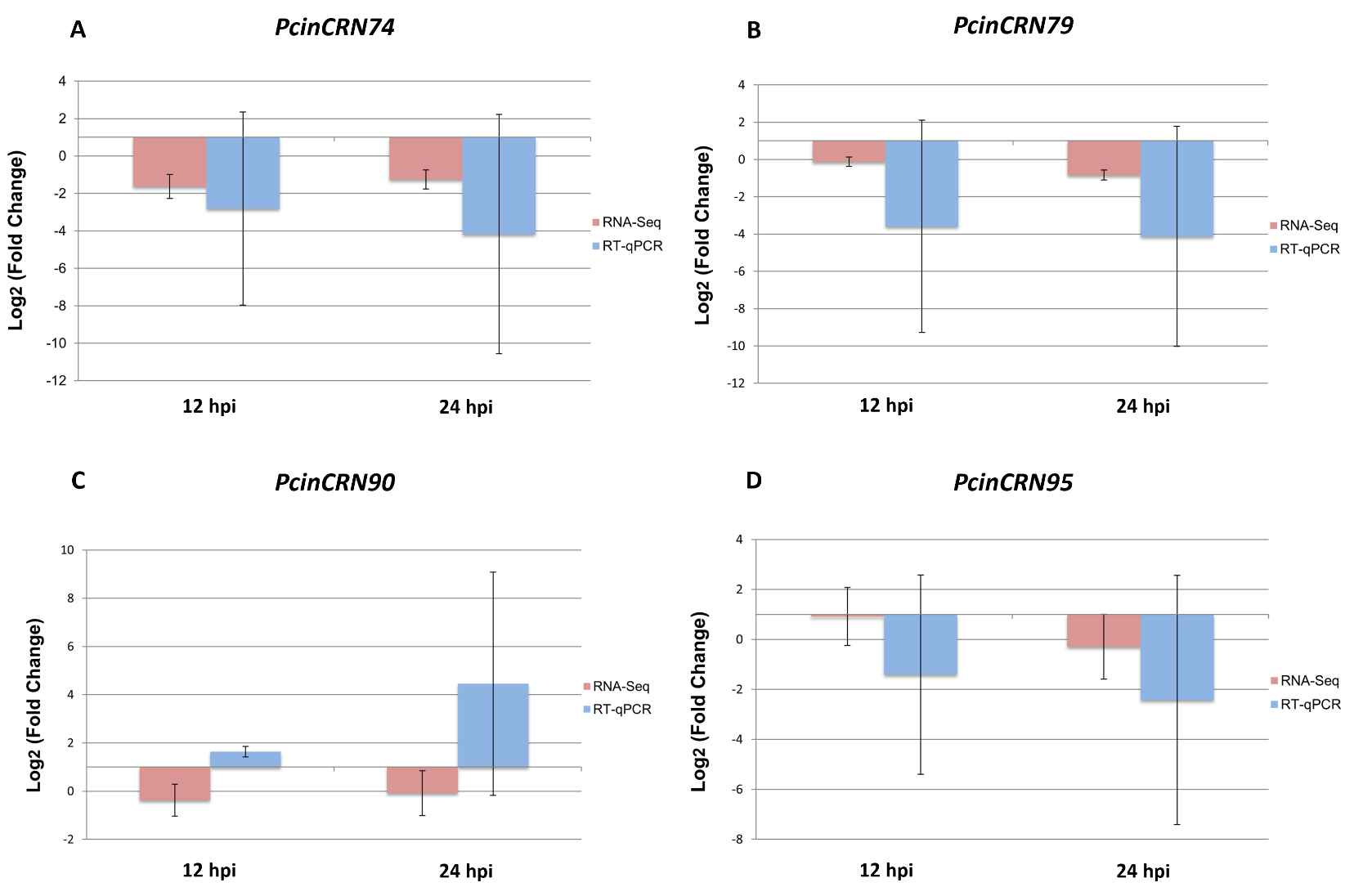
** **
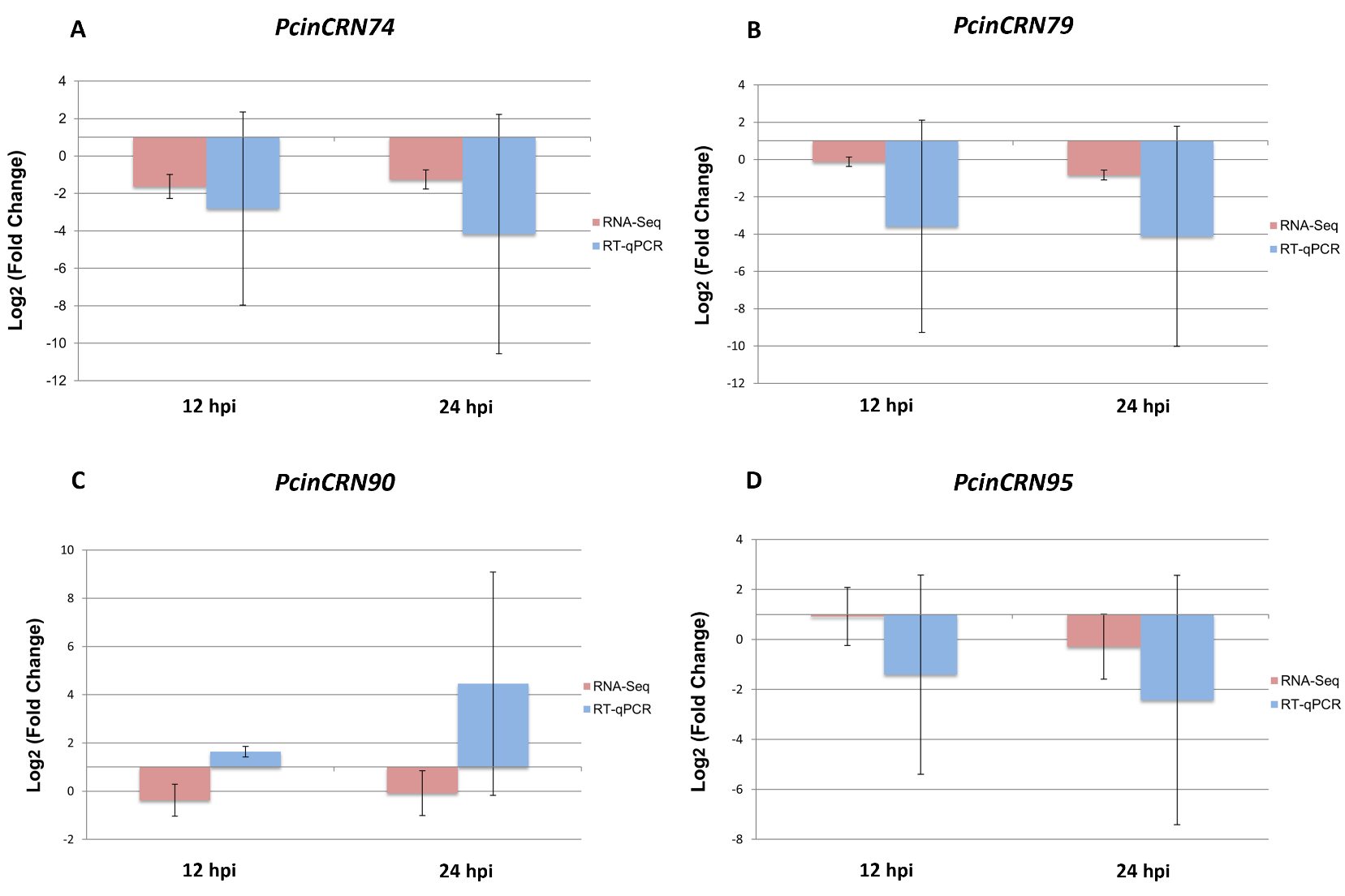
**

**Supplementary Figure 2. Expression validation of selected *Phytophthora cinnamomi crinkling and necrosis (PcinCRN)* effector genes at 12 and 24 hpi compared to mycelial control.** Normalized Log2 (Fold Change) for (A) *PcinCRN74*, (B) *PcinCRN79*, (C) *PcinCRN90*, and (D) *PcinCRN95* were calculated using the method described by Pfaﬄ (2001) (58). The expression of PcinCRNs during infection of R012 avocado rootstock is indicated by vertical bars across two time points using RNA-seq data (red) and RT-qPCR (blue). The samples were compared to *P. cinnamomi* mycelia. SE for each bar is shown.
