# Supplementary material for: Identification of Phytophthora cinnamomi CRN effectors and their roles in manipulating cell death during Persea americana infection
Source: BMC Genomics. 2024 May 2;25:435. doi: 10.1186/s12864-024-10358-3 (PMC11064341; doi:10.1186/s12864-024-10358-3)
Supplement: Supplementary file 5 — Supplementary Material 5 [file 12864_2024_10358_MOESM5_ESM.docx]

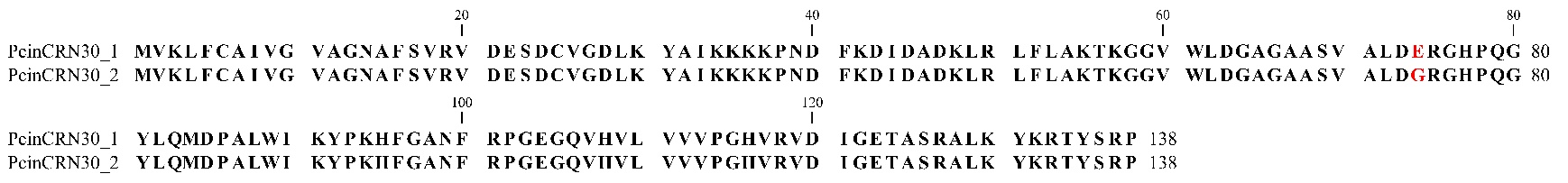


**A**

**B**

**
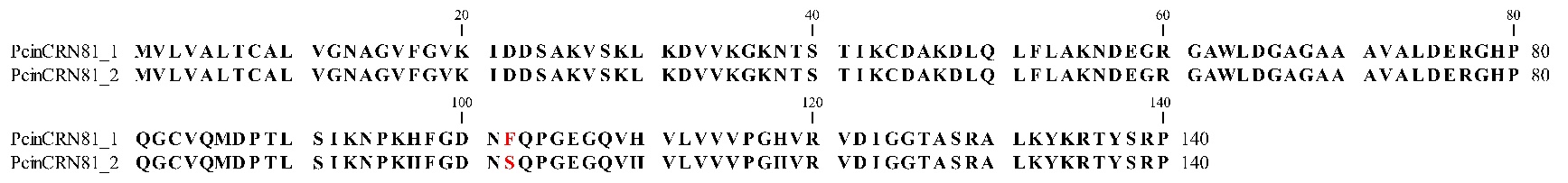
**

**Supplementary Figure 3. Schematic comparing PcinCRN30 and PcinCRN81 protein sequences.** The confirmed amino acid sequences of the *Phytophthora cinnamomi* crinkling and necrosis (PcinCRN) effectors of **(A)** PcinCRN30_1 and PcinCRN30_2, and **(B)** PcinCRN81_1 and PcinCRN81_2 were aligned using CLC Main Workbench with a gap open cost value of 10,0 and a gap extension cost value of 1,0. Both PcinCRN30 and PcinCRN81 have a single nucleotide polymorphism (SNP) change between their variants, which results in a non-synonymous amino acid change indicated in red.
