# Supplementary material for: Identification of Phytophthora cinnamomi CRN effectors and their roles in manipulating cell death during Persea americana infection
Source: BMC Genomics. 2024 May 2;25:435. doi: 10.1186/s12864-024-10358-3 (PMC11064341; doi:10.1186/s12864-024-10358-3)
Supplement: Supplementary file 16 — Supplementary Material 16 [file 12864_2024_10358_MOESM16_ESM.docx]

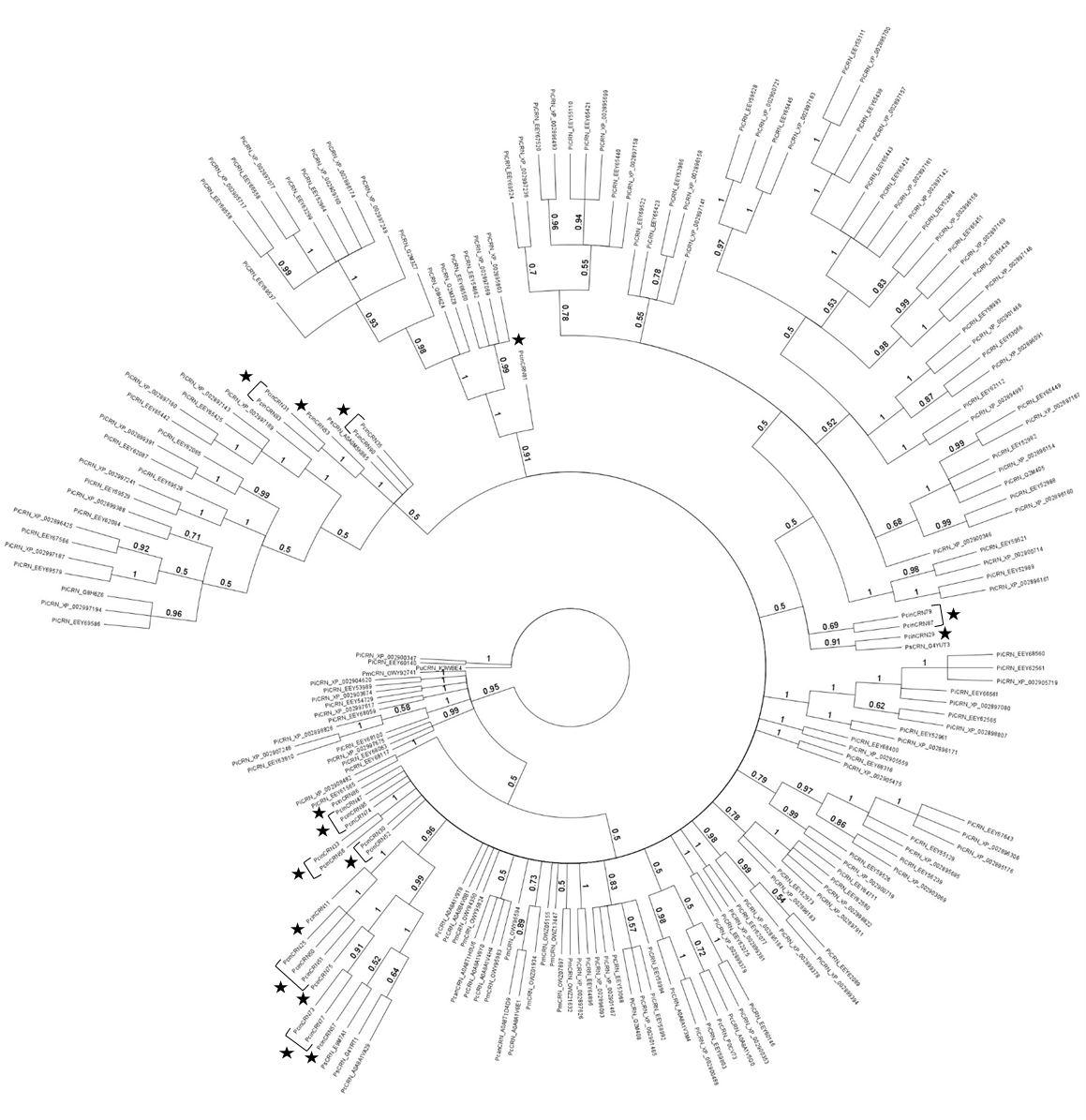
**Supplementary Figure 1. Evolutionary relatedness of identified PcinCRNs to CRNs from other *Phytophthora* spp.** Alignment of predicted N-terminal regions of putative *Phytophthora cinnamomi* crinkling and necrosis (PcinCRN) effectors with the N-terminal regions of CRNs from other *Phytophthora* spp. resulted in the construction of a phylogenetic tree using Bayesian inference analysis. Support for branches is indicated by posterior probability values, which are displayed for each node to the second significant digit with a posterior probability cut-off of < 0.5. Putative PcinCRN effectors proteins are indicated with stars. All 25 PcinCRNs were found to be similar to CRNs from other *Phytophthora* spp.
