# Supplementary material for: Identification of Phytophthora cinnamomi CRN effectors and their roles in manipulating cell death during Persea americana infection
Source: BMC Genomics. 2024 May 2;25:435. doi: 10.1186/s12864-024-10358-3 (PMC11064341; doi:10.1186/s12864-024-10358-3)
Supplement: Supplementary file 17 — Supplementary Material 17 [file 12864_2024_10358_MOESM17_ESM.docx]

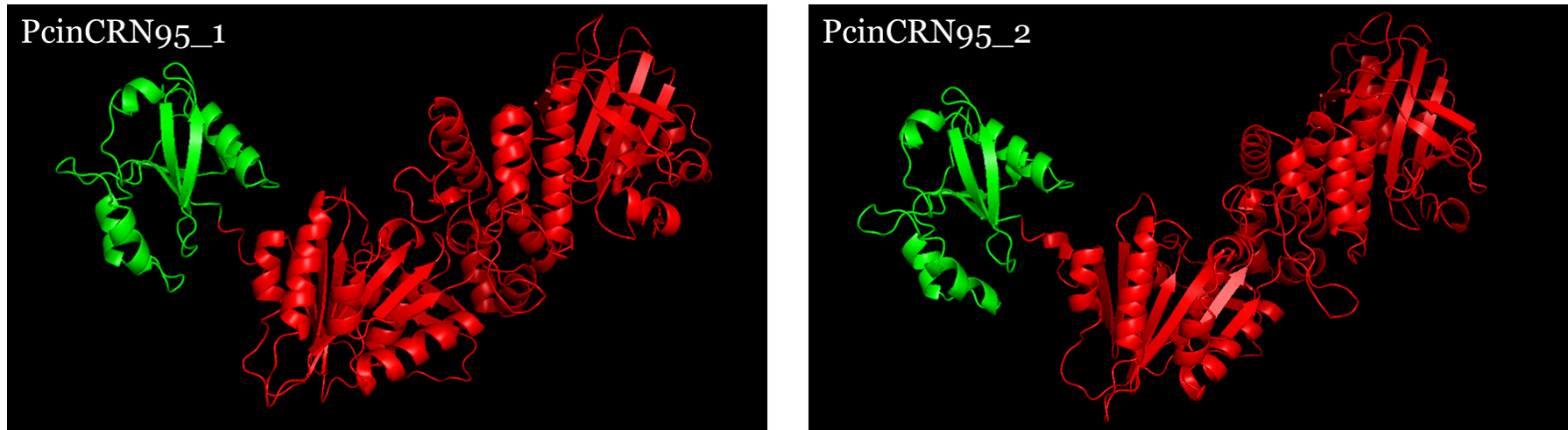


**Supplementary Figure 4. Predicted protein structures of the amino acid sequences encoded by the PcinCRN95 alleles using AlphaFold.** AlphaFold (28, 29) was used to predict the tertiary structure of *Phytophthora cinnamomi* crinkling and necrosis effector protein 95 (PcinCRN95). On a scale from 0 to 100, AlphaFold generated a per-residue confidence metric titled predicted local distance difference test (pLDDT). Protein structure of PcinCRN95_1 (pLDDT = 84.27) and PcinCRN95_2 (pLDDT = 84.89) showed no obvious change in tertiary protein folding between them. A high pLDDT score (> 80) indicates high confidence in the structure of the residue, whereas a low pLDDT score (< 50) may indicate that the residues are in intrinsically disordered protein regions. The predicted tertiary structures were visualised in PyMOL v2.5.5 (Schrödinger, LLC). Green labelled structures represent the N-terminal up until the HVLVXXP motif. Red labelled structures represent the C-terminal.
